# A non-invasive, automated diagnosis of Menière’s disease using radiomics and machine learning on conventional magnetic resonance imaging: A multicentric, case-controlled feasibility study

**DOI:** 10.1007/s11547-021-01425-w

**Published:** 2021-11-25

**Authors:** Marly F. J. A. van der Lubbe, Akshayaa Vaidyanathan, Marjolein de Wit, Elske L. van den Burg, Alida A. Postma, Tjasse D. Bruintjes, Monique A. L. Bilderbeek-Beckers, Patrick F. M. Dammeijer, Stephanie Vanden Bossche, Vincent Van Rompaey, Philippe Lambin, Marc van Hoof, Raymond van de Berg

**Affiliations:** 1 Department of Otolaryngology and Head and Neck Surgery, Maastricht University Medical Center +, Maastricht, The Netherlands; 2 The D-Lab, Department of Precision Medicine, GROW Research Institute for Oncology, Maastricht University, Maastricht, The Netherlands; 3 Research and Development, Oncoradiomics SA, Liege, Belgium; 4 Department of Radiology and Nuclear Medicine, Maastricht University Medical Center, Maastricht, The Netherlands; 5 School for Mental Health and Sciences, Maastricht University, Maastricht, The Netherlands; 6 Department of Otorhinolaryngology, Gelre Hospital, Apeldoorn, The Netherlands; 7 Department of Otorhinolaryngology, Leiden University Medical Center, Leiden, The Netherlands; 8 Department of Radiology, Viecuri Medical Center, Venlo, The Netherlands; 9 Department of Otorhinolaryngology, Viecuri Medical Center, Venlo, The Netherlands; 10 Department of Radiology, Antwerp University Hospital, Antwerp, Belgium; 11 Department of Radiology, AZ St-Jan Brugge-Oostende, Bruges, Belgium; 12 Department of Otorhinolaryngology and Head & Neck Surgery, Antwerp University Hospital, Faculty of Medicine and Health Sciences, University of Antwerp, Antwerp, Belgium

**Keywords:** Menière’s disease, Magnetic resonance imaging, Radiomics, Machine learning

## Abstract

**Purpose:**

This study investigated the feasibility of a new image analysis technique (radiomics) on conventional MRI for the computer-aided diagnosis of Menière’s disease.

**Materials and methods:**

A retrospective, multicentric diagnostic case–control study was performed. This study included 120 patients with unilateral or bilateral Menière’s disease and 140 controls from four centers in the Netherlands and Belgium. Multiple radiomic features were extracted from conventional MRI scans and used to train a machine learning-based, multi-layer perceptron classification model to distinguish patients with Menière’s disease from controls. The primary outcomes were accuracy, sensitivity, specificity, positive predictive value, and negative predictive value of the classification model.

**Results:**

The classification accuracy of the machine learning model on the test set was 82%, with a sensitivity of 83%, and a specificity of 82%. The positive and negative predictive values were 71%, and 90%, respectively.

**Conclusion:**

The multi-layer perceptron classification model yielded a precise, high-diagnostic performance in identifying patients with Menière’s disease based on radiomic features extracted from conventional T2-weighted MRI scans. In the future, radiomics might serve as a fast and noninvasive decision support system, next to clinical evaluation in the diagnosis of Menière’s disease.

**Supplementary Information:**

The online version contains supplementary material available at 10.1007/s11547-021-01425-w.

## Introduction

Menière’s disease (MD) is a multifactorial condition of the inner ear characterized by recurrent episodes of vertigo and fluctuating aural symptoms like hearing loss, aural fullness, and tinnitus. The exact etiology of the disease is unknown. However, MD is strongly associated with the classical histological finding known as endolymphatic hydrops (EH), which is a distention of the endolymphatic compartment of the labyrinth [1]. The consistent finding of EH in temporal bones of patients with MD [2, 3] led to defining EH as the pathological basis of MD. However, it also marked the beginning of a diagnostic challenge, as EH could only be identified post-mortem. As a consequence, MD remained a clinical diagnosis, and different symptom-based classification methods emerged over time [4].

The clinical diagnosis of MD, however, is complicated due to the diverse clinical presentation of the disease, symptom overlap with other etiologies, and the lack of specific biomarkers [5–7]. Therefore, new imaging techniques are under investigation as a MD diagnostic [8, 9]. Nowadays, the most commonly applied technique in clinical practice is delayed gadolinium-enhanced MRI [10, 11]. This technique enables the in-vivo confirmation of EH. Various methods have been proposed to qualitatively and quantitatively assess the endolymphatic space [10]. Most recent developments even allow the fully automatic 3D segmentation and volumetric quantification of the endolymphatic space [12, 13], a significant step towards automatization and standardization of EH assessment on imaging. Nevertheless, EH is not a pathognomic feature to MD. It is observed in various neuro-otologic pathologies as well as in asymptomatic ears [10–12, 14]. The exact relationship between EH and MD and its pathological and clinical relevance are not completely understood. Combining additional image markers with EH, such as the degree of perilymphatic enhancement, seem highly specific for MD [15, 16].

The ongoing developments in delayed gadolinium-enhanced MRI are promising for the role of imaging in the diagnosis of MD. However, there are downsides related to contrast-enhanced MRI. It deals with a delayed scanning protocol. Imaging is performed 24 h after intratympanic (IT) and 4 h after intravenous (IV) administration of gadolinium [9]. Furthermore, IT administration is considered an invasive procedure [9, 11], and IV administration is contraindicated in patients with contrast allergies or renal dysfunction [17]. Although no ototoxicity has been reported [18], adverse effects such as gadolinium deposition in the brain have been observed [17, 19]. Hence, a more efficient, less invasive imaging technique to diagnose MD would be preferable. The concomitant exploration of other potential MD diagnostics remains relevant.

Increasing evidence indicates that diagnostic, prognostic, and predictive information can be extracted from standard-of-care image modalities [20–22]. The process of converting medical images into mineable high-dimensional data by extracting quantitative image features and linking them to clinical outcomes is referred to as radiomics [21, 23]. To analyze such large amounts of image features, machine learning (ML) methods are often used to find patterns in the data.

A preliminary study demonstrated the possible value of radiomics within the diagnosis of MD by detecting differences in image features between patients with MD and controls in conventional MRI scans [24]. To further explore the application of radiomics, the objective of this study was to develop a computer-aided diagnostic tool for MD by using a radiomics approach combined with ML. Its performance and feasibility as a new diagnostic tool for MD were evaluated.

## Materials and methods

### Study design and inclusion

A retrospective, diagnostic case-control study was performed on patients with unilateral and bilateral MD. Medical records in the following centers in the Netherlands and Belgium were searched for eligible subjects:

1. Center A (Maastricht University Medical Center +), The Netherlands
2. Center B (Antwerp University Hospital), Belgium
3. Center C (Apeldoorn Dizziness Center), The Netherlands
4. Center D (VieCuri Hospital Venlo), The Netherlands

For the inclusion of subjects, a conventional MRI scan of the cerebellopontine angle already available from the clinical setting was required. Rough motion artifacts and/or unsharp delineation of the inner ear on MRI was an exclusion criterion. Subjects were enrolled as “patients” when clinically diagnosed by an ENT-specialist as *definite* MD according to the criteria of the American Academy of Otolaryngology-Head and Neck Surgery (AAO-HNS) [25] and/or Barany society (2015) [4]. Both unilateral and bilateral cases of *definite* MD were included. Subjects were enrolled as “controls” when diagnosed by an ENT-specialist with idiopathic asymmetric sensorineural hearing loss. The labyrinth least affected by hearing loss was considered the best representative of a ‘normal’ labyrinth and was included in the study. These patients were chosen as controls since this was a retrospective study and no MRI scans from ‘healthy’ people without any hearing loss were available. Controls were excluded in case of a documented history of vertigo and/or balance disorders.

### Statistical analysis

A Chi-square test of independence was performed for between-group comparisons of gender distribution and independent samples t-test for age distributions. Statistical analyses were carried out using SPSS software version 25.0 (IBM Corp, Armonk, NY).

### Radiomics workflow

**Fig. 1:**
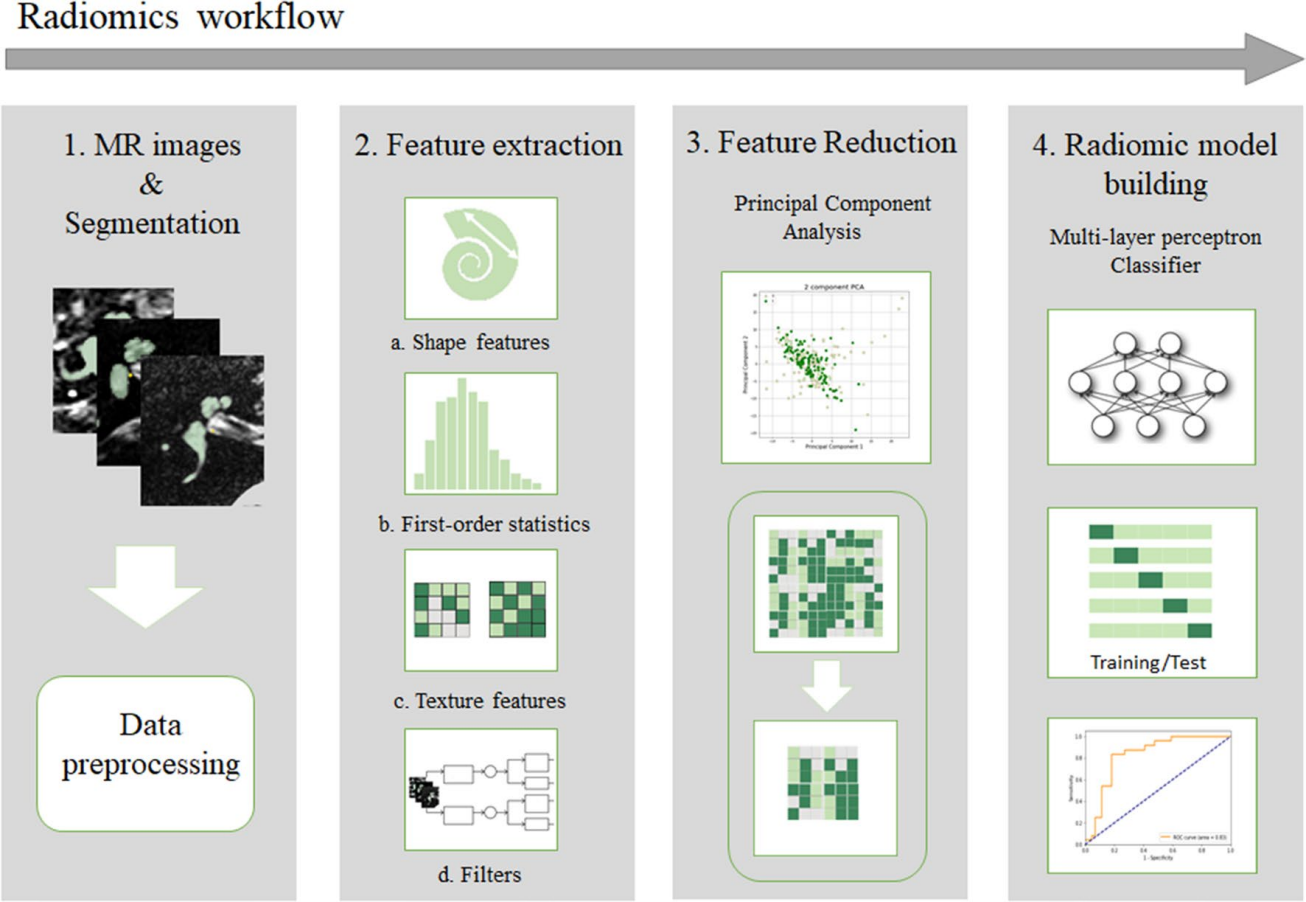
The workflow of Radiomics in this study is graphically presented in four steps. (1) T2-weighted MR images were collected from four different centers in the Netherlands and Belgium and manually segmented. The MR volumes and their corresponding segmentation masks were preprocessed into isotropic voxels. (2) Four types of features (a. Shape features, b. First-order statistic features, c. Texture features, and d. Features extracted after applying different filters) were extracted from the segmented masks. (3) Feature reduction was done by principal component analysis. (4) A multi-layer perceptron classifier was used for radiomic analysis

The radiomics workflow applied in this study consisted of four steps, as illustrated in Fig. 1.

#### MR imaging and segmentation

**Fig. 2:**
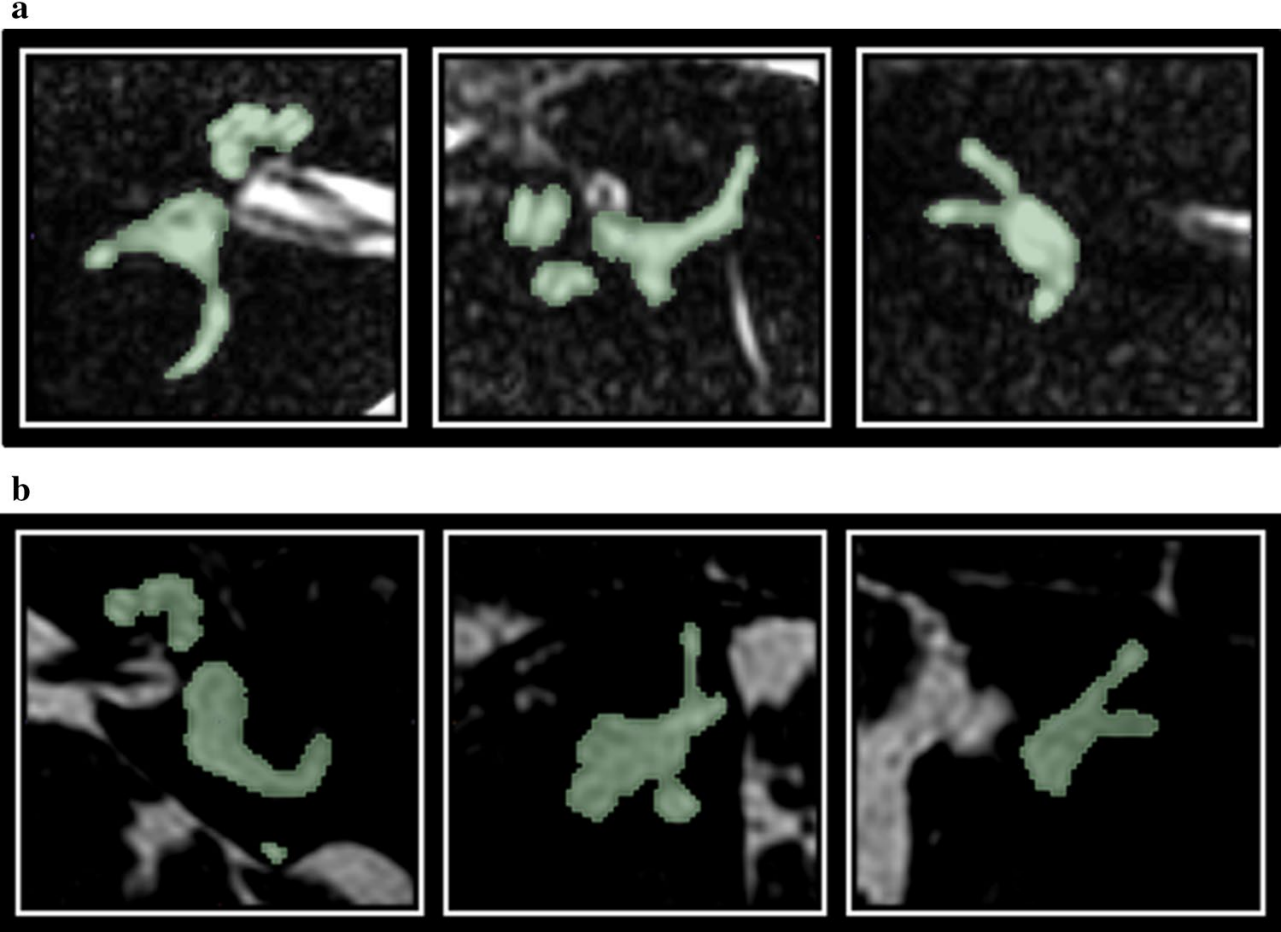
**a** A cropped MR image of a right inner ear of a subject with asymmetric sensorineural hearing loss on the right side. From left to right, the axial, sagittal and coronal planes are presented. The manual segmentation is visualized by the green mask **b** A cropped MR image of a left inner ear of a subject with unilateral Menière’s disease on the left side. From left to right, the axial, sagittal and coronal planes are presented. The manual segmentation is visualized by the green mask

Image acquisition and data anonymization were performed by the local investigators of the four centers. T2-weighted MR images were acquired with center-specific protocols on 1.5 T and 3 T scanners. Scan parameters were not constant between centers, as shown in Table 1 of the supplementary materials. The 3D Slicer 4.8.1 [26] was used to segment the labyrinth from all MRI scans. Two authors (EB, MW) manually segmented all labyrinths using an inbuilt region-growing algorithm (Grow from seeds) [27]. The first author (ML) cleaned the initial dataset and re-segmented the labyrinths in case of missing labels. Examples of manual segmentations are demonstrated in Fig. 2.

**Table 1:** Details of study cohort

Group	n	Center	Menière’s (n)	Controls (n)	Age (years)	Gender (M/F)	Date MRI
Training cohort (74%)	192	A	40	19	60 ± 8	92/67	2004–2017^*^
		B	25	20			
		C	31	57			
		Total	96	96			
Test cohort (26%)	68	A	8	4	61 ± 9	34/25	2004–2017^*^
		B	8	4			
		C	2	18			
		D	6	18			
		Total	24	44			

Demographic details of the study cohorts. N = number of ears, Age is median age with median absolute deviation, * Significant difference between cohorts

The following preprocessing steps were performed [28]. 1) in order to normalize the voxel sizes across the volumes, the MR volumes and their corresponding segmentation masks were resampled to isotropic voxels of length 0.5 mm using cubic spline interpolation. 2) voxel intensities were transformed using Z-score normalization to minimize the influence of contrast or brightness variation among the images. 3) the transformed voxel intensities were discretized using a fixed bin width of 0.5.

#### Feature extraction

In total, 812 radiomic features were extracted from the segmented masks using RadiomX (Oncoradiomics SA, Liège, Belgium). Mathematical descriptions of all features were previously published and presented as Supplemental Material with the permission of the corresponding authors [20, 22, 29].

#### Feature reduction

To reduce the dimensionality of the extracted features, a principal component analysis (PCA) was performed. PCA is an unsupervised, linear dimensionality reduction technique in which small numbers of uncorrelated variables are extracted as “Principal Components” to explain most of the variation in the data in lower dimensions [30, 31]. As a result, essential information holding most of the variation in the data was preserved, and non-essential parts with fewer variations were removed. Ten Principal Components were extracted from the analysis and used to train the model. The inverse PCA was applied to identify the mean contribution of each feature overall principal components in order to predict the most important features. A mean contribution of > 0.7 was chosen to identify 15 features, which had the largest contribution to the PCA.

#### Radiomic model building

A Multi-Layer Perceptron classifier with 500 units in the hidden layer with Adam optimizer at a learning rate of 0.001 was selected for the classification task. The input to the model was the extracted Principal Components. The output layer consisted of a single neuron for each prediction class (patients = 1 and control = 0), which used the Softmax function to output a value between 0 and 1. The output represented the probability of the predicted classes. The regularization method “early stopping” was adopted during training to avoid overfitting the model [32].

Two methods were used for the evaluation of the ML model: a train-test split and k-fold cross-validation.

**Fig. 3:**
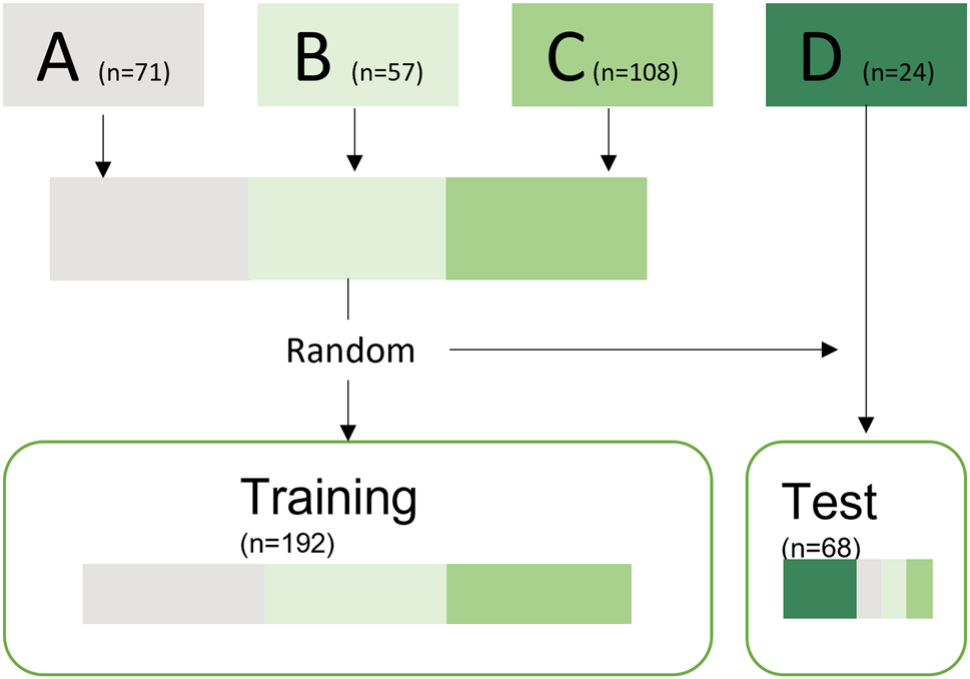
The flowchart of the train-test data split

*Train-test split:* The complete dataset (“patients” and “controls”) was divided into a training and test set of 74% and 26%, respectively. The training set contained images from centers A, B and C. The test set contained images from center D, as an external center. The test set was complemented with randomly selected scans from the other centers (A, B, and C), which were excluded from training. This means no cases were used in both training and testing. The train-test split is graphically presented in Fig. 3.

*K-fold cross-validation:* A 10-fold cross-validation was performed on the complete dataset. The data were randomly split into ten parts. Alternately, nine parts were used for training and one part for testing.

### Outcome measurements

The primary outcomes of this study were accuracy, sensitivity, specificity, positive predictive value, and negative predictive value. The precision (i.e., confidence interval) of each parameter was determined. As additional outcomes, the F1-scores and the Matthews correlation coefficients were reported.

## Results

### Study population

This retrospective study included 120 patients with MD (59 men, 61 women, aged 16–84; median age 59, median absolute difference ± 9) and 140 controls with asymmetric sensorineural hearing loss (67 men, 31 women in 42 controls gender was unknown, aged 6–88; median age 63, median absolute difference ± 7) over four centers. There were 71 labyrinths included from Center A (67.6% MD, 32.4% control), 57 from Center B (59.1% MD, 42.1% control), 108 from Center C (30.6% MD, 69.4% control), and 24 from Center D (35.3% MD, 64.7% control) There was no significant difference in age distribution between the patient and the control group and between the training and test cohort. The proportion of known males versus females did not differ between the test and training cohort. A significant difference in scan date between the training and test cohort was found (independent sample t-test: p = 0.019) with MRI scans of the training cohort being performed on earlier dates. No significant differences in scan date between all patients with MD and controls were found. Details of the training and test cohort are presented in Table 1.

### Principal component analysis

**Fig. 4:**
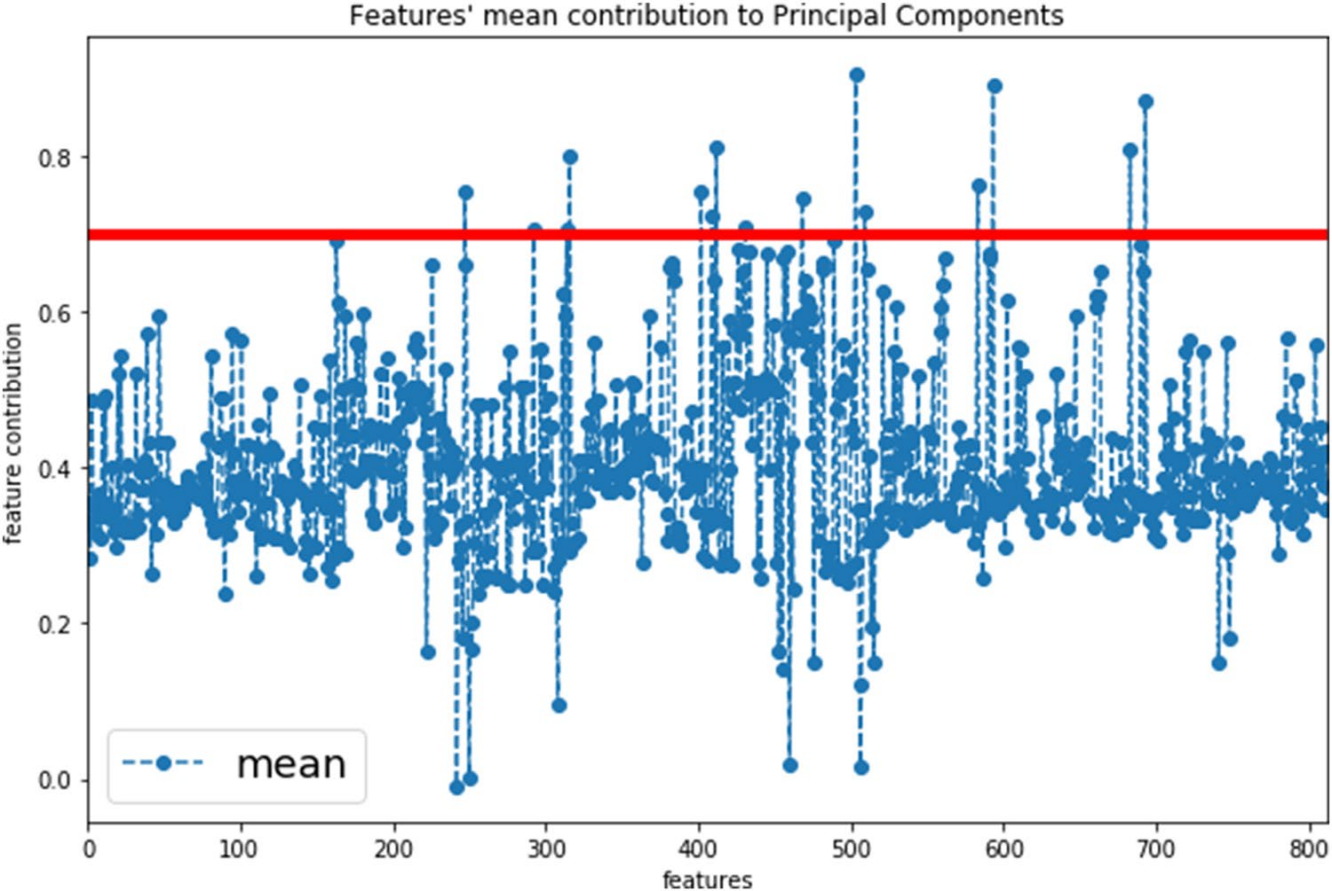
The mean contribution over all principal components aggregated for each feature. The red line indicates the cut-off value (< 0.7) for the most important features that contributed to the PCA

By applying the inverse PCA, the mean contribution of each feature over all principal components is illustrated in Fig. 4. As a result, the features with the most substantial influence on the principal components could be identified. These are presented in Table 2 of the Supplementary Materials.

**Table 2:** Classification performance

	Training cohort	Test cohort	10-fold cross-validation
Patients vs. Controls	96 vs. 96	24 vs. 44	
Accuracy (%)	72.9	82.3	80.0
AUC (95% CI)	80.6 (80.5–81.2)	86.9 (86.6–88.8)	83.6 (77.9–89.3)
Sensitivity (95% CI)	80.2 (80.0–81.1)	83.4 (82.6 -86.9)	78.3 (71.4–85.3)
Specificity (95% CI)	65.6 (65.3–66.3)	81.8 (81.4–83.7)	77.5 (70.5–84.5)
Positive predictive value (95% CI)	70.0 (69.7–70.6)	71.4 (70.4–74.1)	77.6 (69.9–85.4)
Negative predictive value (95% CI)	76.8 (67.5–77.8)	90.0 (89.7 -92.3)	78.4 (70.6–86.3)
F1-scores	0.75	0.77	0.77
MCC	0.46	0.63	0.56

Performance of the multi-layer perceptron classification metric to distinguish MD from healthy controls showing the area under the curve of the Receiver Operating Curve, sensitivity, specificity, positive predictive value, negative predictive value, F1-scores and MCC. The mean F1-scores, and MCC of the 10-fold cross-validation are presented. Abbreviations: *CI* Confidence interval, *AUC* Area under the curve, *MCC* Matthews correlation coefficient

### Machine learning classifier

**Fig. 5:**
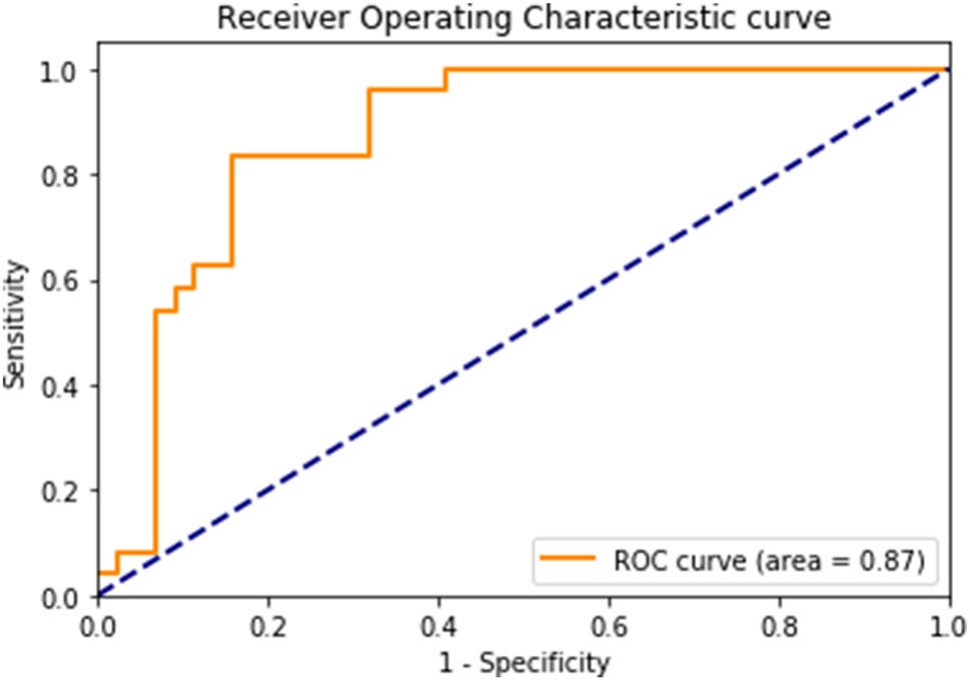
The Receiver Operator Characteristic curve of the test cohort of the multi-layer perceptron classifier

**Fig. 6:**
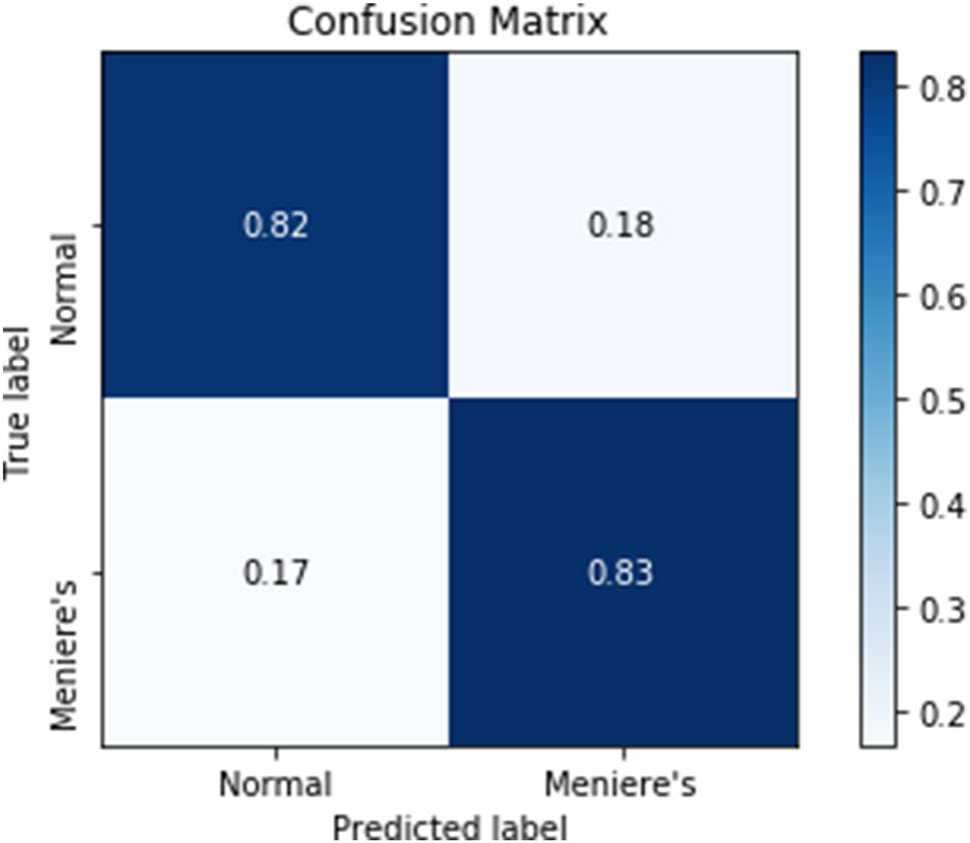
The confusion matrix of the test cohort of the multi-layer perceptron classifier. The true labels are the diagnostic labels after subject inclusion. The predicted labels are the labels predicted by the classifier

The ML model’s performance on classifying patients with MD and controls is demonstrated in Table 2. The classification accuracy of the test set was 82%, with a sensitivity of 83%, specificity of 82%, and AUC of 87%. The positive and negative predictive values were 71% and 90%, respectively. The ROC curve and the confusion matrix are shown in Figs. 5 and 6. The F1-scores and the Matthews correlation coefficients were 0.75 and 0.46 for training and 0.77 and 0.63 for testing, respectively. The results of the 10-fold cross-validation are also presented in Table 2. The mean classification accuracy across the 10-fold was 80%, with a mean sensitivity, specificity, positive predictive value, and negative predictive value of 78%, 77.5%, 78%, and 78%, respectively. The mean AUC was 84%.

## Discussion

This study demonstrated that radiomic features extracted from conventional MRIs could be used to discriminate MD patients from ‘normal’ controls with high accuracy. In a heterogenic, multicentric dataset, the proposed ML model yielded a precise, high-diagnostic performance in identifying patients with MD with an accuracy of 82%. The results are in line with the preliminary study [24], where the feasibility of radiomics was first explored in a small, single-center setup. By integrating ML, the present study proved the value of radiomics in a larger dataset containing heterogeneous images from multiple centers and reported the performance of a diagnostic classification model for MD. The next goal would be to investigate the feasibility of a clinical, prospective application.

### Relevance of radiomics in the diagnosis of MD

Within neuro-otology, radiomics is a very new concept. This study pioneered in developing a computer-aided diagnostic tool for MD by using a radiomic approach. Research on the role of imaging within MD’s diagnosis mainly focuses on the in-vivo visualization of EH on delayed gadolinium-enhanced MRI. One of the main benefits of radiomics is that it can also be applied to MRI scans acquired with no contrast agent. Within the diagnostic workup of MD, these scans are often readily available since MRI is indicated to exclude other causes of asymmetrical hearing loss. Radiomics is not just reserved for centers with specialized radiological expertise on EH. Instead, it could be performed in any center with a (1.5 T or 3 T) MRI scanner, without a delayed scanning protocol, and also in case of allergic reaction to contrast media.

Other studies have also investigated the feasibility of the radiological diagnosis of MD without the use of contrast agents. One publication showed changes in the membranous labyrinth between patients with MD and healthy subjects on 3D CBCT and suggested the usefulness of 3D CBCT imaging for the objective diagnosis of MD [8]. However, the diagnostic value has not been clinically evaluated yet. Three other publications proposed the use of non-contrast T2-weighted MRI for the diagnosis of MD by manually measuring the length and width of the saccule and/or the utricle [33–35]. One of these papers reported the maximum saccular height in healthy volunteers to be 1.6 mm [33]. Another reported a high specificity (95%) but a low sensitivity (63%) for a cut-off value of 1.51 mm for saccular height [34]. Measurements of the absolute utricle area and the utricle-to-vestibule area ratio were also identified as predictors of MD and yielded a sensitivity of 44% and 75% and a specificity of 81% and 53%, respectively. The main disadvantage of these techniques is that only the vestibule and the basal turn of the cochlea were evaluated, while the radiomics method assessed the entire labyrinth. Moreover, human performances on non-contrast MR imaging seem to exhibit lower diagnostic performance compared to radiomics. Prospective studies performing radiomics and vestibular measurements on the same dataset are needed to prove this assumption.

Another benefit of radiomics is that it has the potential to serve as a fully automated diagnostic tool for MD. This study still extracted radiomic features from manually segmented inner ears. Manual segmentation is time-consuming and prone to intra- and inter-observer variability and, therefore, difficult to transpose to routine radiology [36, 37]. However, automatic segmentation of the inner ear has already been made accessible [38]. When integrated with the automatic segmentation of the inner ear, radiomics could be used as a standardized decision support system that might reduce human interference and, therefore, interobserver variability within and between centers. Further research is necessary to evaluate the model’s performance on features extracted from automatically segmented labyrinths.

Eventually, it would be interesting to compare the diagnostic values of automated radiomic analysis with the automatic volumetric assessment of EH on contrast-enhanced MRI.

### Interpretations and mechanism

An essential hypothesis of radiomics is that underlying tissue heterogeneity, associated with cellular and molecular biology, could be captured by the quantitative features extracted from medical images [20, 39]. For instance, several studies have shown a relationship between radiomic features and gene-expression patterns in patients with cancer [22, 40].

The predictive power of the multi-layer perceptron classifier suggested that the extracted radiomic features captured differences in the labyrinth of patients with MD and controls. Hypothetically, these differences could mirror changes in the morphology or perhaps even changes in the molecular biology of the inner ear of patients with MD, allowing an improved understanding of the pathophysiology of the disease. It would be valuable to look into the most discriminative features and identify them as visually perceptible or even visually non-perceptual image biomarkers for MD.

However, one of the main drawbacks of machine learning is the interpretability of the underlying mechanisms on which an ML algorithm generates its output, which is why these methods are often referred to as a “black box” [41]. This study attempted to provide some insights into the “rationale” behind the ML model by identifying the extracted features that contributed most to the principal components and thus, in turn, contributed to a highly predictive model.

An interesting finding was that all of these features were extracted after applying a discrete, one-level three-dimensional wavelet-transform to each MRI [20, 22, 42]. Wavelet-transform effectively decouples textural information by decomposing the original image in low and high frequencies [20, 22, 42]. Wavelet decompositions, however, are mathematically generated. It is not yet possible to explain this mathematical information in a clinical manner [43]. Notwithstanding, it was striking that particularly “intensity” features were identified as the most important features. Lower-signal regions on high-resolution T2-weighted MR images have been previously used as margins for saccular size and morphology, which seems predictive for MD [34, 35]. One could hypothesize that features such as the “minimum gray level” (Stats_min) or the “10^th^ percentile of gray level” (Stats_p10) reflected these lower-signal regions and might be linked to saccular hydrops. However, to date, there are no tools to prove this assumption. It remains unclear which (semantic) features would serve as relevant image markers for MD.

### Limitations

This study has several limitations. Firstly, it is important to recognize that no gold standard test is available to compare the radiomics method with. This retrospective study included patients clinically diagnosed with *definite* MD according to the AAO-HNS criteria [4]. These patients, however, do not represent the full clinical spectrum. After all, patients who do not fulfill these criteria due to the fluctuating aspect of hearing loss (not captured by audiometry) or atypical symptom presentation might be an interesting group to explore with radiomics.

Secondly, the duration and severity of the disease were not considered in this study cohort. Disease duration might alter the morphology of the labyrinth. For example, the severity of EH in patients with MD seems to increase with the duration of the disease [44, 45]. Perhaps, the disease duration could alter the composition of the endolymphatic fluid as well. The patient cohort in this study probably contained patients with different disease stages. Early disease stages might be challenging to recognize since important image features were not yet significantly present. Adding clinical information about disease duration would probably have improved the model’s performance.

Thirdly, the proposed radiomics analysis relied on manual segmentation, which is a limitation to overcome in future studies. Inter- and intra-observer variability in manual segmentation was assumed but not assessed in the present study. Although the vestibular aqueduct could contain valuable information for the diagnosis of MD [46], this structure was not specifically considered in the study due to difficulties in manual segmentation. All are relevant points to keep in mind for future studies.

Fourthly, the study dealt with a small dataset consisting of MRI scans from four independent centers. In the absence of sufficient datapoints, it was inevitable to divide the data into just a training and test set, where ideally, a third split would be made to provide an unbiased evaluation of the model. Cross-validation was adopted to help reduce biased results [47]. In addition, this study included an independent dataset (Center D) in testing to better detect overfitting. Overfitting happens when the model learns details and noise to fit the training data with high accuracy but fails to perform on a new set of data [32, 48]. The addition of an external set in testing helps to apply early stopping when the model starts to overfit on the training dataset (i.e., when the training loss decreases and validation loss starts to increase). Due to the small size of the training dataset, overfitting could not be completely avoided. The risk of overfitting was further contained by diversifying the training data. This was done by acquiring data from four different centers and by manually segmenting the labyrinth by three different observers. Therefore, the model should be more generalizable for differences in center-specific scan parameters and inter-reader segmentations.

Lastly, the heterogeneities in voxel spacing and slice thickness between the images were handled by isotropic resampling. This could have induced noise due to interpolations. The results of this study are encouraging as a proof-of-concept. Additional studies with more training data and validation on multiple external datasets with images from different MRI scanners are needed before definitively claiming the model’s generalizability. Exploring the use of convolutional neural networks for the direct extraction of deep features from the raw MRI [49] might also improve the diagnostic accuracy and the generalizability of the model.

### Clinical implications and future perspectives

Radiomics is a new imaging analysis technique that could enable standardized, non-invasive, and widely accessible diagnostic care for patients with MD. The output of the multi-layer perceptron classifier provides a value between 0 and 1, which represents the probability of the predicted classes. This will allow clinicians to interpret the probability of having MD based on the features extracted from MRIs together with the clinical profile of the patients. Before clinical implementation, further research should focus on three main aspects: 1) Integrating auto-segmentation of the inner ear with radiomics analyses, 2) Further validation of the classification model on a larger training dataset and external validation on multiple external datasets, and 3) Evaluation of the diagnostic value of radiomics as an equivalent of contrast-enhanced MR imaging.

The potential role of radiomics, for now, is mainly to aid the clinical diagnosis of MD as a clinical decision support system. However, there lie more perspectives in the future for radiomics. In the current study, only patients with MD were included. However, radiomics might apply to other labyrinthine disorders as well. One study indicated that cochlea CT image features could be useful biomarkers for predicting sensorineural hearing loss in patients treated with chemoradiotherapy for head and neck cancer [50]. It would be valuable to study the relationship between radiomic features and hearing loss in different causes of sensorineural hearing loss. Performing radiomics in more patients with different disorders will eventually allow a comparison between the general vestibular population and a healthy population. Performing a cluster analysis might reveal a reclassification of vestibular disorders based on similarities in radiomic signatures. Compared to symptoms, radiomic signatures might better classify vestibular disorders.

## Conclusion

The automated extraction of radiomic features from conventional MRI scans proved to be valuable to discriminate patients with Menière’s disease and ‘normal’ controls. In the current study, the machine learning-based multi-layer perceptron network yielded a precise, high-diagnostic performance in identifying patients with Menière’s disease with an accuracy of 82%. In the future, radiomics could possibly be implemented as a fast, non-invasive, and accurate decision support system, next to clinical evaluation, in the diagnostic trajectory of Menière’s disease.

## Supplementary Information

Below is the link to the electronic supplementary material.

Supplementary file1 (DOC 858 kb)
