# Supplementary material for: A non-invasive, automated diagnosis of Menière’s disease using radiomics and machine learning on conventional magnetic resonance imaging: A multicentric, case-controlled feasibility study
Source: Radiol Med. 2021 Nov 25;127(1):72–82. doi: 10.1007/s11547-021-01425-w (PMC8795017; doi:10.1007/s11547-021-01425-w)
Supplement: Supplementary file 1 — Supplementary file1 (DOC 858 kb) [file 11547_2021_1425_MOESM1_ESM.doc]

**Supplementary Materials**

**A non-invasive, automated diagnosis of Menière’s disease using Radiomics and Machine Learning on conventional magnetic resonance imaging**

A multicentric, case-controlled feasibility study

Marly F.J.A. van der Lubbe^1^, Akshayaa Vaidyanathan^2,3^, Marjolein de Wit^1^, Elske L. van den Burg^1^, Alida A. Postma^4,5^, Tjasse D. Bruintjes^6,7^, Monique A.L. Bilderbeek-Beckers^8^, Patrick F.M. Dammeijer^9^, Stephanie Vanden Bossche^10,11^, Vincent Van Rompaey^12^, Philippe Lambin^2^, Marc van Hoof^1^, Raymond van de Berg^1,5^

**Author information**

1. Department of Otolaryngology and Head and Neck Surgery, Maastricht University Medical Center, Maastricht, The Netherlands. Email: [marly.lubbe@mumc.nl](mailto:marly.lubbe@mumc.nl)
2. The D-Lab, department of Precision Medicine, GROW research institute for Oncology, Maastricht University, Maastricht, The Netherlands
3. Research and Development, Oncoradiomics SA, Liege, Belgium
4. Department of Radiology and Nuclear Medicine, Maastricht University Medical Center, Maastricht, The Netherlands
5. School for Mental Health and Sciences, Maastricht University, Maastricht, The Netherlands
6. Department of Otorhinolaryngology, Gelre Hospital, Apeldoorn, The Netherlands
7. Department of Otorhinolaryngology, Leiden University Medical Center, Leiden, The Netherlands
8. Department of Radiology, Viecuri Medical Center, Venlo, The Netherlands
9. Department of Otorhinolaryngology, Viecuri Medical Center, Venlo, The Netherlands
10. Department of Radiology, Antwerp University Hospital, Antwerp, Belgium
11. Department of Radiology, AZ St-Jan Brugge-Oostende, Bruges, Belgium
12. Department of Otorhinolaryngology and Head & Neck Surgery, Antwerp University Hospital, Faculty of Medicine and Health Sciences, University of Antwerp, Antwerp, Belgium

***Corresponding author:** Marly van der Lubbe, Vissersmaas 3c 6211EV Maastricht, marly.lubbe@mumc.nl

**Table 1.** Scan parameters of the included MRI scans

| Vendor | Sequence | Repetition time (ms) | Echo time (ms) | Slice thickness (mm) | Pixel spacing (mm) | Echo train length | Percentage  (%) |
| --- | --- | --- | --- | --- | --- | --- | --- |
| **1.5 T MRI scanners** | | | | | | |  |
| *Siemens Aera* | T2 SPACE | 1200-1400 | 148-266 | 0.5-0.7 | 0-0.6 | 5.58-88 | 35 |
| *Siemens Avanto* | T2 3D-CISS  T2 SPACE | 1200 | 265 | 1 | 0 | 87 | 2.3 |
| *Siemens Sonata* | T2 3D-CISS  3D T2 TSE | 8.9-1500 | 4.5-269 | 0.5-0.6 | 0.4-0.5 | 1-37 | 1.9 |
| *Siemens Symphony* | 3D T2 TSE | 1500 | 301 | 0.5 | 0.47 | 37 | 4.2 |
| *GE Genesis Signa* | 3D FIESTA | 4.6 | 1.6 | 0.8 | 0.4 | 0 | 16.5 |
| *Philips Intera* | 3D T2 DRIVE | 1500 | 169-183 | 0.6 | 0.3 | 40 | 4.2 |
| **3 T MRI scanners** | | | | | | |  |
| *Siemens Prisma* | 3D T2 TSE  3D T2 SPACE | 1000 | 126-130 | 0.5 | 0.47-0.52 | 62-64 | 3.5 |
| *Siemens Skyra* | 3D T2 TSE | 1000 | 127-129 | 0.5 | 0.47-0.52 | 63-64 | 1.5 |
| *Siemens Trio* | T2 3D-CISS  3D T2 TSE | 6.4-1400 | 3.2-320 | 0.4-0.8 | 0.4-0.5 | 1-37 | 6.5 |
| *Philips Achieva* | 3D COCHLEA | 1500-2000 | 193-200 | 0.8-1 | 0.4-0.5 | 40-59 | 23.9 |

**Table 2.** Most important features

| **Rank** | **Feature** | **Description** | **Category** |
| --- | --- | --- | --- |
| 1 | 'Wavelet_HHL_Stats_min' | “Minimum” | First-order Grey-level statistics |
| 2 | 'Wavelet_HLH_IVH_AIRV_90' | “Absolute Intensity above Relative volume threshold” | First-order Grey-level statistics |
| 3 | 'Wavelet_HLH_Stats_p10’ | “10th percentile ” | First-order Grey-level statistics |
| 4 | 'Wavelet_HLH_Stats_qcod' | “Quartile coefficient of dispersion ” | Intensity histogram |
| 5 | 'Wavelet_HLL_Stats_cov' | “ Coefficient of variance ” | Intensity histogram |
| 6 | 'Wavelet_HLL_Stats_min', | “Minimum” | First-order Grey-level statistics |
| 7 | 'Wavelet_HLL_Stats_qcod' | “Quartile coefficient of dispersion ” | Intensity histogram |
| 8 | 'Wavelet_LHH_GLCM_sumEntro' | “Sum Entropy” | Grey-Level Co-Occurrence Matrix based |
| 9 | 'Wavelet_LHH_IH_minGrad' | “Minimum histogram gradient” | Intensity histogram |
| 10 | 'Wavelet_LHH_Stats_cov' | “Coefficient of variance” | Intensity histogram |
| 11 | 'Wavelet_LHH_Stats_min' | “Minimum” | First-order Grey-level statistics |
| 12 | 'Wavelet_LHL_Stats_cov' | “Coefficient of variance” | Intensity histogram |
| 13 | 'Wavelet_LHL_Stats_qcod' | “Quartile coefficient of dispersion ” | First-order Grey-level statistics |
| 14 | 'Wavelet_LLH_Stats_cov' | “Coefficient of variance” | Intensity histogram |
| 15 | 'Wavelet_LLH_Stats_qcod' | “Quartile coefficient of dispersion” | Intensity histogram |

The following features were identified to have the largest contribution to the principal components (cut-off > 0.7).

# Feature descriptions

*First-order features* were obtained from the intensity histograms using first-order statistics, including intensity mean, median, maximum, minimum, range, energy, entropy, kurtosis, and skewness. *Shape* *features* were obtained from the 3D shape of delineated volumes. *Texture features* were obtained from the spatial distribution of fractal dimensions and voxel intensities using 6 texture matrices, including grey-level co-occurrence (GLCM), gray-level distance-zone (GLDZM), grey-level run-length (GLRM), grey-level size-zone (GLSZM), neighboring grey-level dependence (NGLDM) and neighborhood grey-tone difference matrix (NGTDM). Furthermore, 3D wavelet, Laplacian, and Gaussian filters were applied on the original images to extract additional *first-order,* *shape,* and *texture* features.

## First-order Grey-level statistics

First-order Grey-level statistics describe the distribution of Grey-values within the volume. Let 𝑋 denote the three dimensional image matrix with 𝑁 voxels, 𝑃 the first order histogram, 𝑃(𝑖) the fraction of voxels with intensity level 𝑖 and 𝑁_𝑙_ the number of discrete intensity levels.

### 1. Energy

𝑁

𝑒𝑛𝑒𝑟𝑔𝑦 = ∑ 𝑋(𝑖)^2^

𝑖=1 Energy is also known as the sum of squares.

### 2. Entropy

𝑁_𝑙_

𝑒𝑛𝑡𝑟𝑜𝑝𝑦 = ∑ 𝑃(𝑖) log_2_ 𝑃(𝑖)

𝑖=1

### 3. Kurtosis

𝑘𝑢𝑟𝑡𝑜𝑠𝑖𝑠
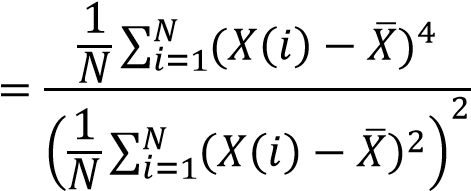


where 𝑋̅ is the mean of 𝑋.

### 4. Maximum

The maximum intensity value of 𝑋.

𝑚𝑎𝑥𝑖𝑚𝑢𝑚 = max⁡(𝑋)

### 5. Mean

The mean Grey-value of 𝑋**.**

𝑁

𝑚𝑒𝑎𝑛 = 𝑋(𝑖)

1

𝑁

∑

𝑖=1

### 6. Mean absolute deviation

The mean of the absolute deviations of all voxel intensities around the mean intensity value.

𝑁

𝑚𝑒𝑎𝑛⁡𝑎𝑏𝑠𝑜𝑙𝑢𝑡𝑒⁡𝑑𝑒𝑣𝑖𝑎𝑡𝑖𝑜𝑛
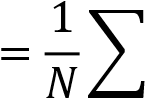
|𝑋(𝑖) − 𝑋|

𝑖=1

where 𝑋̅ is the mean of 𝑋.

**7. Median**

The sample median of 𝑋, or the 50^th^ percentile of 𝑋.

### 8. Minimum

The minimum intensity value of 𝑋.

𝑚𝑖𝑛𝑖𝑚𝑢𝑚 = min(𝑋)

### 9. Range

The range of intensity values of 𝑋.

𝑟𝑎𝑛𝑔𝑒 = max(𝑋) − min(𝑋)

### 10. Root mean square (RMS)

The quadratic mean, or the square root of the mean of squares of all voxel intensities.

√∑𝑁𝑖 𝑋(𝑖)2 𝑅𝑀𝑆 =

𝑁

### 11. Skewness

𝑠𝑘𝑒𝑤𝑛𝑒𝑠𝑠
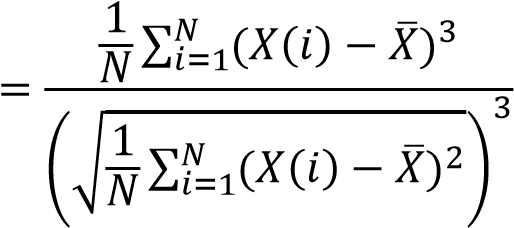


where 𝑋̅ is the mean of 𝑋.

### 12. Standard deviation

𝑁 1⁄2

𝑠𝑡𝑎𝑛𝑑𝑎𝑟𝑑⁡𝑑𝑒𝑣𝑖𝑎𝑡𝑖𝑜𝑛


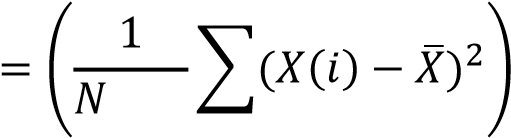
− 1

𝑖=1

where 𝑋̅ is the mean of 𝑋.

### 13. Robust mean absolute deviation

The mean absolute deviation (0) of only those voxels in 𝑋 with a Grey-value between the 10^th^ and 90^th^ percentile.

1. **10^th^ percentile**

The 10^th^ percentile of 𝑋, a robust alternative to the minimum Grey-value (8).

1. **90^th^ percentile**

The 90^th^ percentile of 𝑋, a robust alternative to the maximum Grey-value (4).

1. **Interquartile range**

The interquartile range is defined as the 75^th^ minus the 25^th^ percentile of 𝑋.

### 17. Uniformity

𝑁_𝑙_

𝑢𝑛𝑖𝑓𝑜𝑟𝑚𝑖𝑡𝑦 = ∑ 𝑃(𝑖)^2^

𝑖=1

### 18. Variance

𝑁

𝑣𝑎𝑟𝑖𝑎𝑛𝑐𝑒


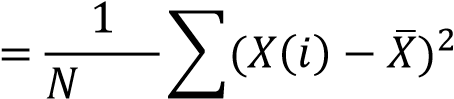
− 1

𝑖=1

where 𝑋̅ is the mean of 𝑋. Variance is the square of the standard deviation (12).

## Geometric features

Geometric features describe the shape and size of the volume of interest. Let 𝑉⁡be the volume and 𝐴 the surface area of the volume of interest. Let 𝑁 be the total number of voxels, 𝑋 = {𝑋⃗_1_, 𝑋⃗_2_, … , 𝑋⃗_𝑁_} the set of N Cartesian coordinate vectors and 𝐼 = {𝐼_1_, 𝐼_2_, … , 𝐼_𝑁_} the corresponding intensity values. **19. Asphericity**

1 𝐴^3^
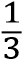


𝑎𝑠𝑝ℎ𝑒𝑟𝑖𝑐𝑖𝑡𝑦 = (36𝜋 𝑉_2_) − 1

### 20. Centroid distance

The centroid distance is the Euclidean distance between the geometric centroid (𝐶_𝑔_) and the centroid weighing each voxel by its intensity value (𝐶_𝑖_). The centroid distance is a measure of how close the high intensity values are to the geometric center.

𝑁

1

𝐶_𝑔_ = ∑ 𝑋⃗_𝑖_

𝑁

𝑖=1

∑𝑁𝑖=1 𝐼𝑋⃗𝑖

𝐶_𝑖_ = ∑^𝑁^ 𝐼

_𝑖_=1

𝑐𝑒𝑛𝑡𝑟𝑜𝑖𝑑⁡𝑑𝑖𝑠𝑡𝑎𝑛𝑐𝑒 = ‖𝐶_𝑔_ − 𝐶_𝑖_‖

### 21. Compactness 1

Compactness is a measure of how much the volume resembles a sphere, as described by Aerts et al. [2].

𝑉

𝑐𝑜𝑚𝑝𝑎𝑐𝑡𝑛𝑒𝑠𝑠⁡
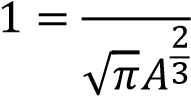


### 22. Compactness 2

𝑉2

𝑐𝑜𝑚𝑝𝑎𝑐𝑡𝑛𝑒𝑠𝑠⁡2 = 36𝜋 𝐴_3_

### 23. Compactness 3

𝑉

𝑐𝑜𝑚𝑝𝑎𝑐𝑡𝑛𝑒𝑠𝑠⁡
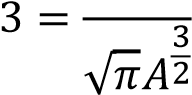


A dimensionless alternative to Compactness (21), as described by Aerts et al. [2]

### 24. Maximum diameter

The maximum diameter is the largest pairwise difference between voxels on the surface of the volume, in 3D and for each plane separately. The following diameters are calculated:

**24.1.** The maximum three-dimensional tumor diameter.

**24.2.** The maximum two-dimensional diameter of all transversal planes.

**24.3.** The maximum two-dimensional diameter of all sagittal planes.

**24.4.** The maximum two-dimensional diameter of all coronal planes.

### 25. Major axis length

Axis lengths are measures of the extent of the volume along its three principle axis. Principle component analysis (PCA) on the x, y and z coordinates of all voxels within the volume is used to determine the three orthogonal eigenvectors and corresponding eigenvalues (𝜆_𝑚𝑎𝑥_, 𝜆_𝑚𝑖𝑛𝑜𝑟_, 𝜆_𝑚𝑖𝑛_). The major axis length is the largest eigenvalue (𝜆_𝑚𝑎𝑥_) as determined by PCA.

1. **Minor axis length**

The largest eigenvalue (𝜆_𝑚𝑖𝑛𝑜𝑟_) as determined by PCA.

1. **Least axis length**

The smallest eigenvalue (𝜆_𝑚𝑖𝑛_) as determined by PCA.

### 28. Elongation

𝜆𝑚𝑖𝑛𝑜𝑟

𝑒𝑙𝑜𝑛𝑔𝑎𝑡𝑖𝑜𝑛 =

𝜆𝑚𝑎𝑥

### 29. Flatness

𝜆𝑚𝑖𝑛

𝑓𝑙𝑎𝑡𝑛𝑒𝑠𝑠 =

𝜆𝑚𝑎𝑥

### 30. Spherical disproportion [3]

Spherical disproportion is a measure of how much the volume resembles a sphere.

𝐴

𝑠𝑝ℎ𝑒𝑟𝑖𝑐𝑎𝑙⁡𝑑𝑖𝑠𝑝𝑟𝑜𝑝𝑜𝑟𝑡𝑖𝑜𝑛 = 4𝜋𝑅_2_

Where 𝐴 is the surface area and 𝑅⁡is the radius of a sphere with the same volume as the tumor, obtained through:

𝑅 =

√

3

𝑉

4

𝜋

3

### 31. Sphericity [3]

Sphericity is a measure of how much the volume resembles a sphere.

𝜋
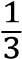
(6𝑉)
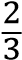
 (36𝜋𝑉^2^)
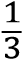


𝑠𝑝ℎ𝑒𝑟𝑖𝑐𝑖𝑡𝑦 = =

𝐴 𝐴

### 32. Surface area

The surface area is calculated by triangulation (i.e. dividing the surface into connected triangles, which define the isosurface enclosing the volume) and is defined as:

𝑁

𝑠𝑢𝑟𝑓𝑎𝑐𝑒⁡𝑎𝑟𝑒𝑎
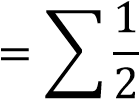
 |𝑎_𝑖_𝑏_𝑖_×𝑎_𝑖_𝑐_𝑖_|

𝑖=1

Where 𝑁 is the total number of triangles covering the surface and 𝑎, 𝑏 and 𝑐 are edge vectors of the triangles.

### 33. Surface to volume ratio

𝐴

𝑠𝑢𝑟𝑓𝑎𝑐𝑒⁡𝑡𝑜⁡𝑣𝑜𝑙𝑢𝑚𝑒⁡𝑟𝑎𝑡𝑖𝑜 = 𝑉

### 34. Volume

The volume is defined as the number of voxels within the volume multiplied by the voxel volume.

𝑣𝑜𝑙𝑢𝑚𝑒 = 𝑁𝑣

Where 𝑣 is the volume of a single voxel.

## Grey-Level Co-Occurrence Matrix based features

Grey level co-occurrence matrix (GLCM) based features, as originally described by Haralick et al [4]. A normalized GLCM is defined as 𝑃(𝑖, 𝑗; 𝛿, 𝛼), a matrix with size 𝑁_𝑔_×𝑁_𝑔_ describing the second-order joint probability function of an image, where the (𝑖, 𝑗)th element represents the number of times the combination of intensity levels 𝑖 and 𝑗 occur in two pixels in the image, that are separated by a distance of 𝛿 pixels in direction 𝛼, and 𝑁_𝑔_ is the maximum discrete intensity level in the image. Let:

𝑃(𝑖, 𝑗) be the normalized (i.e. ∑ 𝑃(𝑖, 𝑗) = 1) co-occurrence matrix, generalized for any 𝛿 and 𝛼 ,


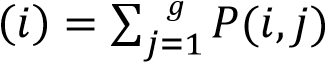
𝑁

𝑝_𝑥_,

𝑝_𝑦_
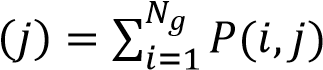
,

𝜇_𝑥_ be the mean of 𝑝_𝑥_, where 𝜇_𝑥_
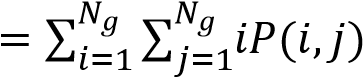


𝜇_𝑦_ be the mean of 𝑝_𝑦_, where 𝜇_𝑦_
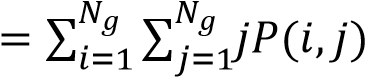


𝜎_𝑥_ be the standard deviation of 𝑝_𝑥_, where 𝜎_𝑥_^2^
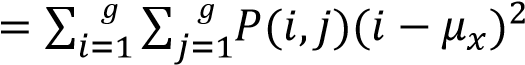
𝑁 𝑁

𝜎_𝑦_ be the standard deviation of 𝑝_𝑦_, where 𝜎
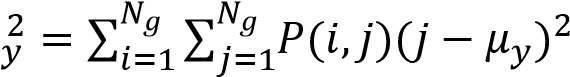


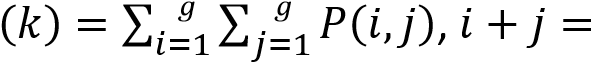
 𝑁 𝑁

𝑝_𝑥+𝑦_𝑘, 𝑘 = 2,3, … ,2𝑁_𝑔_,

𝑝_𝑥−𝑦_
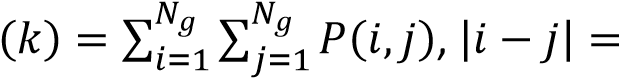
 𝑘, 𝑘 = 0,1, … , 𝑁_𝑔_ − 1,

𝐻𝑋𝑌
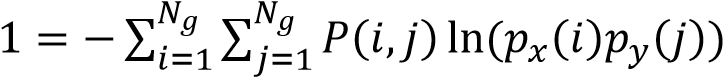
 ⁡,


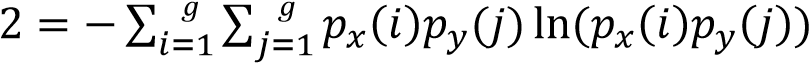
 𝑁 𝑁

𝐻𝑋𝑌.

𝐻𝑋 = ∑ 𝑝_𝑥_ ln(𝑝_𝑥_)

𝐻𝑌 = ∑ 𝑝_𝑦_ ln(𝑝_𝑦_)

### 35. Average (𝝁)


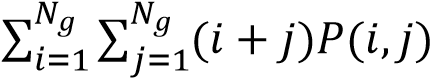


𝑎𝑣𝑒𝑟𝑎𝑔𝑒⁡(𝜇) =

2

Note that for a symmetrical GLCM, 𝜇 = 𝜇_𝑥_ = 𝜇_𝑦_.

### 36. Autocorrelation

𝑁𝑔 𝑁𝑔

𝑎𝑢𝑡𝑜𝑐𝑜𝑟𝑟𝑒𝑙𝑎𝑡𝑖𝑜𝑛 = ∑ ∑ 𝑖𝑗𝑃(𝑖, 𝑗)

𝑖=1 𝑗=1

### 37. Cluster Prominence

𝑁𝑔 𝑁𝑔

4

𝑐𝑙𝑢𝑠𝑡𝑒𝑟⁡𝑝𝑟𝑜𝑚𝑖𝑛𝑒𝑛𝑐𝑒 = ∑ ∑[𝑖 + 𝑗 − 𝜇_𝑥_ − 𝜇_𝑦_] 𝑃(𝑖, 𝑗)

𝑖=1 𝑗=1

### 38. Cluster Shade

𝑁𝑔 𝑁𝑔

3

𝑐𝑙𝑢𝑠𝑡𝑒𝑟⁡𝑠ℎ𝑎𝑑𝑒 = ∑ ∑[𝑖 + 𝑗 − 𝜇_𝑥_ − 𝜇_𝑦_] 𝑃(𝑖, 𝑗)

𝑖=1 𝑗=1

### 39. Cluster Tendency

𝑁𝑔 𝑁𝑔

2

𝑐𝑙𝑢𝑠𝑡𝑒𝑟⁡𝑡𝑒𝑛𝑑𝑒𝑛𝑐𝑦 = ∑ ∑[𝑖 + 𝑗 − 𝜇_𝑥_ − 𝜇_𝑦_] 𝑃(𝑖, 𝑗)

𝑖=1 𝑗=1

### 40. Contrast [5]

𝑁𝑔 𝑁𝑔 𝑁𝑔−1

𝑐𝑜𝑛𝑡𝑟𝑎𝑠𝑡 = ∑ ∑|𝑖 − 𝑗|^2^𝑃(𝑖, 𝑗) = ∑ 𝑘^2^ 𝑝_𝑥−𝑦_(𝑘)

𝑖=1 𝑗=1 𝑘=0

### 41. Correlation


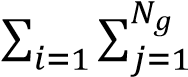
𝑁𝑔

𝑖𝑗𝑃(𝑖, 𝑗) − 𝜇_𝑥_𝜇_𝑦_

𝑐𝑜𝑟𝑟𝑒𝑙𝑎𝑡𝑖𝑜𝑛 =

𝜎𝑥𝜎𝑦

### 42. Difference Average (𝝁_𝒙−𝒚_)

𝑁_𝑔_−1

𝑑𝑖𝑓𝑓𝑒𝑟𝑒𝑛𝑐𝑒⁡𝑎𝑣𝑒𝑟𝑎𝑔𝑒⁡(𝜇_𝑥−𝑦_) = ∑ 𝑘𝑝_𝑥−𝑦_

𝑘=0

### 43. Difference Entropy

𝑁_𝑔_−1

𝑑𝑖𝑓𝑓𝑒𝑟𝑒𝑛𝑐𝑒⁡𝑒𝑛𝑡𝑟𝑜𝑝𝑦 = − ∑ 𝑃_𝑥−𝑦_(𝑖) log_2_[𝑃_𝑥−𝑦_(𝑖)]

𝑖=0

### 44. Difference Variance

𝑁_𝑔_−1

𝑑𝑖𝑓𝑓𝑒𝑟𝑒𝑛𝑐𝑒⁡𝑣𝑎𝑟𝑖𝑎𝑛𝑐𝑒 = ∑ (𝑖 − 𝜇_𝑥−𝑦_)^2^𝑃_𝑥−𝑦_(𝑖)

𝑖=0

### 45. Dissimilarity

𝑁𝑔 𝑁𝑔

𝑑𝑖𝑠𝑠𝑖𝑚𝑖𝑙𝑎𝑟𝑖𝑡𝑦 = ∑ ∑|𝑖 − 𝑗|𝑃(𝑖, 𝑗)

𝑖=1 𝑗=1

### 46. Energy [6]

𝑁𝑔 𝑁𝑔

𝑒𝑛𝑒𝑟𝑔𝑦 = ∑ ∑[𝑃(𝑖, 𝑗)]^2^

𝑖=1 𝑗=1

This feature is also called Angular Second Moment (ASM) and Uniformity [5].

### 47. Entropy (H)

𝑁𝑔 𝑁𝑔

𝑒𝑛𝑡𝑟𝑜𝑝𝑦⁡(𝐻) = − ∑ ∑ 𝑃(𝑖, 𝑗) log_2_[𝑃(𝑖, 𝑗)]

𝑖=1 𝑗=1

### 48. Homogeneity 1

𝑁𝑔 𝑁𝑔

ℎ𝑜𝑚𝑜𝑔𝑒𝑛𝑒𝑖𝑡𝑦⁡1 =
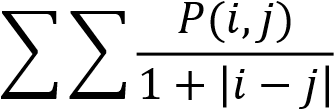


𝑖=1 𝑗=1

This feature is also called Inverse Difference [5].

### 49. Homogeneity 2 [6]

𝑁𝑔 𝑁𝑔


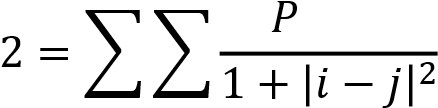
(𝑖, 𝑗)

ℎ𝑜𝑚𝑜𝑔𝑒𝑛𝑒𝑖𝑡𝑦⁡

𝑖=1 𝑗=1

This feature is also called Inverse Difference Moment [5].

### 50. Informational measure of correlation 1 (IMC1)

𝐻 − 𝐻𝑋𝑌1

𝐼𝑀𝐶1 =

max{𝐻𝑋, 𝐻𝑌}

Where 𝐻 is the entropy (47).

### 51. Informational measure of correlation 2 (IMC2)

𝐼𝑀𝐶
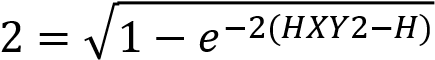


Where 𝐻 is the entropy (47).

### 52. Inverse Difference Moment Normalized (IDMN)

𝑁𝑔 𝑁𝑔

𝑃(𝑖, 𝑗)

𝐼𝐷𝑀𝑁 = ∑ ∑

|𝑖 − 𝑗|2

𝑖=1 𝑗=1 1 + ( 𝑁_𝑔2_ )

### 53. Inverse Difference Normalized (IDN)

𝑁𝑔 𝑁𝑔

𝑃(𝑖, 𝑗)

𝐼𝐷𝑁 = ∑ ∑

|𝑖 − 𝑗|

𝑖=1 𝑗=1 1 + ( _𝑁𝑔_ )

### 54. Inverse variance

𝑁𝑔 𝑁𝑔

𝑃(𝑖, 𝑗)

𝑖𝑛𝑣𝑒𝑟𝑠𝑒⁡𝑣𝑎𝑟𝑖𝑎𝑛𝑐𝑒 = ∑ ∑ |𝑖 − 𝑗|_2_ , 𝑖 ≠ 𝑗

𝑖=1 𝑗=1

### 55. Maximal Correlation Coefficient

𝑚𝑎𝑥𝑖𝑚𝑎𝑙⁡𝑐𝑜𝑟𝑟𝑒𝑙𝑎𝑡𝑖𝑜𝑛⁡𝑐𝑜𝑒𝑓𝑓𝑖𝑐𝑖𝑒𝑛𝑡 = √second⁡largest⁡eigenvalue⁡of⁡𝑄

𝑁𝑔

𝑄


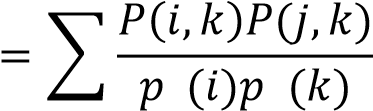
 𝑥 𝑦

𝑘=1

**56. Maximum Probability**

𝑚𝑎𝑥𝑖𝑚𝑢𝑚⁡𝑝𝑟𝑜𝑏𝑎𝑏𝑖𝑙𝑖𝑡𝑦 = max{𝑃(𝑖, 𝑗)}

### 57. Sum average (SA)

2𝑁_𝑔_

𝑠𝑢𝑚⁡𝑎𝑣𝑒𝑟𝑎𝑔𝑒⁡(𝑆𝐴) = ∑[𝑖𝑃_𝑥+𝑦_(𝑖)]

𝑖=2

### 58. Sum entropy

2𝑁_𝑔_

𝑠𝑢𝑚⁡𝑒𝑛𝑡𝑟𝑜𝑝𝑦 = − ∑ 𝑃_𝑥+𝑦_(𝑖) log_2_[𝑃_𝑥+𝑦_(𝑖)]

𝑖=2

### 59. Sum variance

2𝑁_𝑔_

𝑠𝑢𝑚⁡𝑣𝑎𝑟𝑖𝑎𝑛𝑐𝑒 = ∑(𝑖 − 𝑆𝐴)^2^𝑃_𝑥+𝑦_(𝑖)

𝑖=2

### 60. Variance (sum of squares)

𝑁𝑔 𝑁𝑔

𝑣𝑎𝑟𝑖𝑎𝑛𝑐𝑒 = ∑ ∑(𝑖 − 𝜇)^2^𝑃(𝑖, 𝑗)

𝑖=1 𝑗=1

## Grey-Level Run-Length matrix based features

Grey-level run-length matrix (GLRLM) based features, as described by Galloway et al. [7]. Run length metrics quantify Grey level runs in an image. A Grey level run is defined as the length in number of pixels, of consecutive pixels that have the same Grey level value. In a Grey level run length matrix 𝑝(𝑖, 𝑗|𝜃), the (𝑖, 𝑗)th element describes the number of times 𝑗 a Grey level 𝑖 appears consecutively in the direction specified by 𝜃. Let:

𝑝(𝑖, 𝑗) be the (𝑖, 𝑗)th entry in the given run-length matrix 𝑝, generalized for any direction 𝜃,

𝑁_𝑔_ the number of discrete intensity values in the image,

𝑁_𝑟_ the maximum run length,

𝑁_𝑠_ the total number of runs, where 𝑁_𝑠_
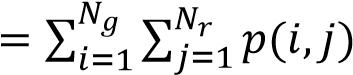
,

𝑝_𝑟_ the sum distribution of the number of runs with run length 𝑗, where 𝑝_𝑟_
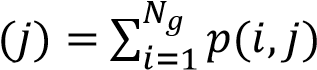
,

𝑝_𝑔_ the sum distribution of the number of runs with Grey level 𝑖, where 𝑝_𝑔_
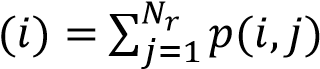
,

𝑁_𝑝_ the number of voxels in the image, where 𝑁_𝑝_ = ^∑𝑁^_𝑗=_^𝑟^_1_ 𝑗𝑝_𝑟_,

𝑝_𝑛_(𝑖, 𝑗) the normalized run-length matrix, where 𝑝_𝑛_(𝑖, 𝑗) ^= 𝑝(^_𝑁_^𝑖^𝑠^,𝑗)^,

𝜇_𝑟_ the mean run length, where 𝜇_𝑟_
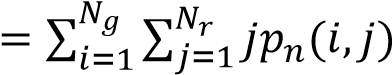
,

𝜇_𝑔_ the mean Grey level, where 𝜇_𝑔_
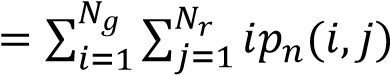
.

### 61. Short Run Emphasis (SRE)

𝑁_𝑟_

1 𝑝_𝑟_

𝑆𝑅𝐸 = 𝑁_𝑠_ ∑ 𝑗_2_

𝑗=1

### 62. Long Run Emphasis (LRE)

𝑁_𝑟_

𝐿𝑅𝐸
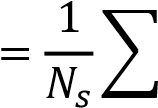
𝑗^2^𝑝_𝑟_

𝑗=1

### 63. Grey Level Non-Uniformity (GLN)

𝑁𝑔

𝐺𝐿𝑁𝑝_𝑔_^2^


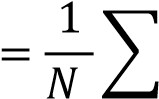
𝑠

𝑖=1

### 64. Grey Level Non-Uniformity Normalized (GLNN)

𝑁𝑔

𝐺𝐿𝑁𝑁
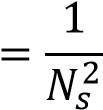
 ∑ 𝑝_𝑔_^2^

𝑖=1

### 65. Run Length Non-Uniformity (RLN)

𝑁_𝑟_

𝑅𝐿𝑁
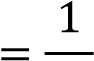
 ∑ 𝑝_𝑟_^2^

𝑁_𝑠_

𝑗=1

### 66. Run Length Non-Uniformity Normalized (RLNN)

𝑁_𝑟_

1

𝑅𝐿𝑁𝑁 = 𝑁_𝑠2_ ∑ 𝑝_𝑟_2

𝑗=1

### 67. Run Percentage (RP)

𝑁

𝑅𝑃 = _𝑠_

𝑁_𝑝_

### 68. Low Grey Level Run Emphasis (LGRE)

𝑁𝑔

1 𝑝_𝑔_

𝐿𝐺𝑅𝐸 = 𝑁_𝑠_ ∑ 𝑖_2_

𝑖=1

### 69. High Grey Level Run Emphasis (HGRE)

𝑁𝑔


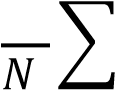
 𝐻𝐺𝑅𝐸 = 1 𝑖2𝑝𝑔

𝑠

𝑖=1

### 70. Short Run Low Grey Level Emphasis (SRLGE)

𝑁𝑔 𝑁𝑟

1 𝑝(𝑖, 𝑗)

𝑆𝑅𝐿𝐺𝐸 = 𝑁 ∑ ∑ 𝑖_2𝑗_2

𝑠

𝑖=1 𝑗=1

### 71. Short Run High Grey Level Emphasis (SRHGE)

𝑁𝑔 𝑁𝑟

1 𝑝(𝑖, 𝑗)𝑖^2^

𝑆𝑅𝐻𝐺𝐸 ∑ ∑ 2


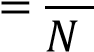
 𝑠 𝑗

𝑖=1 𝑗=1

### 72. Long Run Low Grey Level Emphasis (LRLGE)

𝑁𝑔 𝑁

𝑟 2 𝐿𝑅𝐿𝐺𝐸
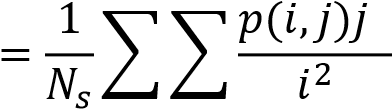


𝑖=1 𝑗=1

### 73. Long Run High Grey Level Emphasis (LRHGE)

𝑁𝑔 𝑁𝑟

𝐿𝑅𝐻𝐺𝐸
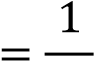
 ∑ ∑ 𝑝(𝑖, 𝑗)𝑖^2^𝑗^2^

𝑁_𝑠_

𝑖=1 𝑗=1

### 74. Grey level variance (GLV)

𝑁𝑔 𝑁𝑟

𝐺𝐿𝑉 = ∑ ∑(𝑖 − 𝜇_𝑔_)^2^𝑝_𝑛_(𝑖, 𝑗)

𝑖=1 𝑗=1

### 75. Run length variance (RLV)

𝑁𝑔 𝑁𝑟

𝑅𝐿𝑉 = ∑ ∑(𝑗 − 𝜇_𝑟_)^2^𝑝_𝑛_(𝑖, 𝑗)

𝑖=1 𝑗=1

### 76. Run entropy (RE) [8]

𝑁𝑔 𝑁𝑟

𝑅𝐸 = − ∑ ∑ 𝑝_𝑛_(𝑖, 𝑗|𝜃)log_2_[ 𝑝_𝑛_(𝑖, 𝑗)]

𝑖=1 𝑗=1

## Grey-Level size-zone matrix based features

Grey-level size-zone matrix (GLSZM) based features, as described by Thibault et al. [9, 10]. A Grey level size-zone matrix describes the amount of homogeneous connected areas within the volume, of a certain size and intensity. The (𝑖, 𝑗)th entry of the GLSZM 𝑝(𝑖, 𝑗) is the number of connected areas of Grey-level (i.e. intensity value) 𝑖 and size 𝑗. GLSZM features therefore describe homogeneous areas within the tumor volume, describing tumor heterogeneity at a regional scale [11]. Let:

𝑝(𝑖, 𝑗) be the (𝑖, 𝑗)th entry in the given GLSZM 𝑝,

𝑁_𝑔_ the number of discrete intensity values in the image,

𝑁_𝑧_ the size of the largest, homogeneous region in the volume of interest,

𝑁_𝑠_ the total number of homogeneous regions (zones), where 𝑁_𝑠_
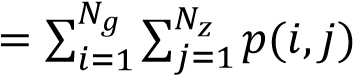
,

𝑝_𝑧_ the sum distribution of the number of zones with size 𝑗, where 𝑝_𝑧_
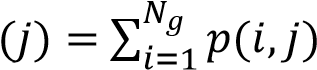
,

𝑝_𝑔_ the sum distribution of the number of zones with Grey level 𝑖, where 𝑝_𝑔_
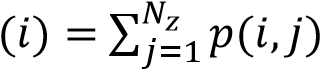
,

𝑁_𝑝_ the number of voxels in the image, where 𝑁_𝑝_ = ^∑𝑁^_𝑗=_^𝑧^_1_ 𝑗𝑝_𝑧_,

𝑝(𝑖,𝑗)

𝑝_𝑛_(𝑖, 𝑗) the normalized size-zone matrix, where 𝑝_𝑛_(𝑖, 𝑗)⁡= _𝑁_𝑠 ,

𝜇_𝑧_ the mean zone size, where 𝜇_𝑧_
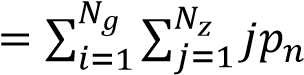
(𝑖, 𝑗|𝜃),

𝜇_𝑔_ the mean Grey level, where 𝜇_𝑔_
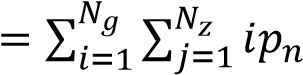
(𝑖, 𝑗|𝜃).

### 77. Small area Emphasis (SAE)

𝑁_𝑧_

1 𝑝_𝑧_

𝑆𝐴𝐸 = 𝑁_𝑠_ ∑ 𝑗_2_

𝑗=1

### 78. Large area Emphasis (LAE)

𝑁_𝑧_

𝐿𝐴𝐸
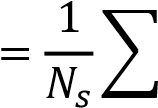
𝑗^2^𝑝_𝑧_

𝑗=1

### 79. Intensity Non-Uniformity (IN)

𝑁𝑔

𝐼𝑁𝑝_𝑔_^2^


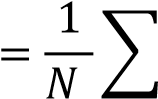
𝑠

𝑖=1

### 80. Intensity Non-Uniformity Normalized (INN)

𝑁𝑔

𝐼𝑁𝑁
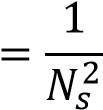
 ∑ 𝑝_𝑔_^2^

𝑖=1

### 81. Size-zone Non-Uniformity (SZN)

𝑁_𝑧_

𝑆𝑍𝑁
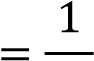
 ∑ 𝑝_𝑧_^2^

𝑁_𝑠_

𝑗=1

### 82. Size-zone Non-Uniformity Normalized (SZNN)

𝑁_𝑧_

1

𝑆𝑍𝑁𝑁 = 𝑁_𝑠2_ ∑ 𝑝_𝑧_2

𝑗=1

### 83. Zone Percentage (ZP)

𝑁

𝑍𝑃 = _𝑠_

𝑁_𝑝_

### 84. Low intensity Emphasis (LIE)

𝑁𝑔

1 𝑝_𝑔_

𝐿𝐼𝐸 = 𝑁_𝑠_ ∑ 𝑖_2_

𝑖=1

### 85. High intensity Emphasis (HIE)

𝑁𝑔


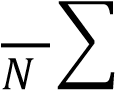
 𝐻𝐼𝐸 = 1 𝑖2𝑝𝑔

𝑠

𝑖=1

### 86. Low intensity small area Emphasis (LISAE)

𝑁𝑔 𝑁𝑧

1 𝑝(𝑖, 𝑗)

𝐿𝐼𝑆𝐴𝐸 = 𝑁 ∑ ∑ 𝑖_2𝑗_2

𝑠

𝑖=1 𝑗=1

### 87. High intensity small area Emphasis (HISAE)

𝑁𝑔 𝑁𝑧

1 𝑝(𝑖, 𝑗)𝑖^2^

𝐻𝐼𝑆𝐴𝐸 ∑ ∑ 2


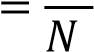
 𝑠 𝑗

𝑖=1 𝑗=1

### 88. Low intensity large area Emphasis (LILAE)

𝑁𝑔 𝑁


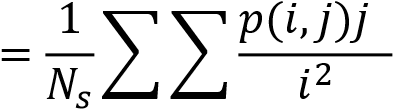
 𝑧 2

𝐿𝐼𝐿𝐴𝐸

𝑖=1 𝑗=1

### 89. High intensity large area Emphasis (HILAE)

𝑁𝑔 𝑁𝑧

𝐻𝐼𝐿𝐴𝐸
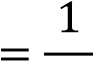
 ∑ ∑ 𝑝(𝑖, 𝑗)𝑖^2^𝑗^2^

𝑁_𝑠_

𝑖=1 𝑗=1

### 90. Intensity variance (IV)

𝑁𝑔 𝑁𝑧

𝐼𝑉 = ∑ ∑(𝑖 − 𝜇_𝑔_)^2^𝑝_𝑛_(𝑖, 𝑗)

𝑖=1 𝑗=1

### 91. Size-zone variance (SZV)

𝑁𝑔 𝑁𝑧

𝑆𝑍𝑉 = ∑ ∑(𝑗 − 𝜇_𝑧_)^2^𝑝_𝑛_(𝑖, 𝑗)

𝑖=1 𝑗=1

### 92. Zone entropy (ZE)

𝑁𝑔 𝑁𝑧

𝑍𝐸 = ∑ ∑ 𝑝_𝑛_(𝑖, 𝑗)log_2_[ 𝑝_𝑛_(𝑖, 𝑗)]

𝑖=1 𝑗=1

## Grey-Level distance-zone matrix based features

Grey-level distance-zone matrix (GLDZM) based features, as described by Thibault et al. [12]. A Grey level distance-zone matrix describes the amount of homogeneous connected areas within the volume, of a certain intensity and distance to the shape border. The shape border is defined by 6-connectedness in 3D (i.e. a voxel is on the border, if at least one face is exposed). In contrast to the original definition by Thibault et al. [12], the minimum distance to the border is 1, instead of 0 (i.e. voxels on the border have a distance of 1), to allow for correct feature calculations. The (𝑖, 𝑗)th entry of the GLDZM 𝑝(𝑖, 𝑗) is the number of connected areas of Grey-level (i.e. intensity value) 𝑖 and minimum distance 𝑗 to the shape border. GLSZM features therefore describe the radial distribution of homogeneous areas within the tumor volume. Let:

𝑝(𝑖, 𝑗) be the (𝑖, 𝑗)th entry in the given GLDZM 𝑝,

𝑁_𝑔_ the number of discrete intensity values in the image,

𝑁_𝑑_ the largest distance of a homogeneous region in the volume of interest to the shape border, 𝑁_𝑠_ the total number of homogeneous regions (zones), where 𝑁_𝑠_
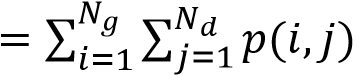
,


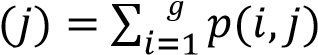
𝑁

𝑝_𝑑_ the sum distribution of the number of zones with distance 𝑗, where 𝑝_𝑧_,

𝑝_𝑔_ the sum distribution of the number of zones with Grey level 𝑖, where 𝑝_𝑔_
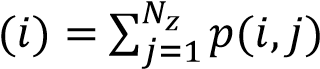
, 𝑁_𝑝_ the number of voxels in the image, where 𝑁_𝑝_
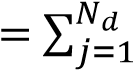
 𝑗𝑝_𝑑_,

𝑝(𝑖,𝑗)

𝑝_𝑛_(𝑖, 𝑗) the normalized size-zone matrix, where 𝑝_𝑛_(𝑖, 𝑗)⁡= _𝑁_𝑠 ,

𝜇_𝑑_ the mean distance, where 𝜇_𝑑_
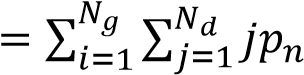
(𝑖, 𝑗|𝜃),

𝜇_𝑔_ the mean Grey level, where 𝜇_𝑔_
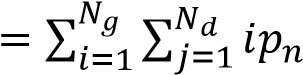
(𝑖, 𝑗|𝜃).

### 93. Small distance Emphasis (SDE)

𝑁𝑑

1 𝑝_𝑑_

𝑆𝐷𝐸 = 𝑁_𝑠_ ∑ 𝑗_2_

𝑗=1

### 94. Large distance Emphasis (LDE)

𝑁𝑑

𝐿𝐷𝐸
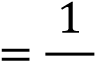
 ∑ 𝑗^2^𝑝_𝑑_

𝑁_𝑠_

𝑗=1

### 95. Intensity Non-Uniformity (IN)

𝑁𝑔

𝐼𝑁𝑝_𝑔_^2^


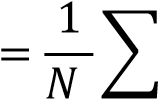
𝑠

𝑖=1

### 96. Intensity Non-Uniformity Normalized (INN)

𝑁𝑔

𝐼𝑁𝑁
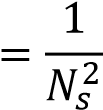
 ∑ 𝑝_𝑔_^2^

𝑖=1

### 97. Distance-zone Non-Uniformity (DZN)

𝑁𝑑

𝐷𝑍𝑁
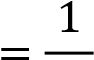
 ∑ 𝑝_𝑑_^2^

𝑁_𝑠_

𝑗=1

### 98. Distance-zone Non-Uniformity Normalized (DZNN)

𝑁𝑑

1

𝐷𝑍𝑁𝑁 = 𝑁_𝑠2_ ∑ 𝑝_𝑑_2

𝑗=1

### 99. Zone Percentage (ZP)

𝑁

𝑍𝑃 = _𝑠_

𝑁_𝑝_

### 100. Low intensity Emphasis (LIE)

𝑁𝑔

1 𝑝_𝑔_ 𝐿𝐼𝐸 ∑ 𝑖_2_


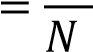
𝑠

𝑖=1

### 101. High intensity Emphasis (HIE)

𝑁𝑔


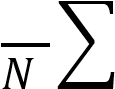
 𝐻𝐼𝐸 = 1 𝑖2𝑝𝑔

𝑠

𝑖=1

### 102. Low intensity small distance Emphasis (LISDE)

𝑁𝑔 𝑁𝑑

1 𝑝(𝑖, 𝑗) 𝐿𝐼𝑆𝐷𝐸
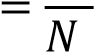
𝑠 ∑ ∑ 𝑖_2𝑗_2

𝑖=1 𝑗=1

### 103. High intensity small distance Emphasis (HISDE)

𝑁𝑔 𝑁

𝑑 2 𝐻𝐼𝑆𝐷𝐸
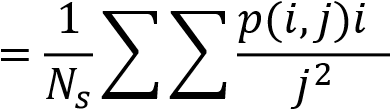


𝑖=1 𝑗=1

### 104. Low intensity large distance Emphasis (LILDE)

𝑁𝑔 𝑁

𝑑 2

𝐿𝐼𝐿𝐷𝐸
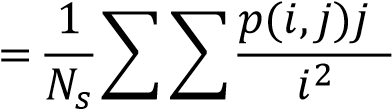


𝑖=1 𝑗=1

### 105. High intensity large distance Emphasis (HILDE)

𝑁

𝑔 𝑁𝑑

𝐻𝐼𝐿𝐷𝐸𝑝(𝑖, 𝑗)𝑖^2^𝑗^2^


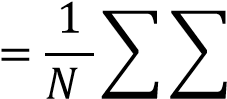
𝑠

𝑖=1 𝑗=1

### 106. Intensity variance (IV)

𝑁𝑔 𝑁𝑑

𝐼𝑉 = ∑ ∑(𝑖 − 𝜇_𝑔_)^2^𝑝_𝑛_(𝑖, 𝑗)

𝑖=1 𝑗=1

### 107. Distance-zone variance (DZV)

𝑁𝑔 𝑁𝑑

𝑆𝑍𝑉 = ∑ ∑(𝑗 − 𝜇_𝑑_)^2^𝑝_𝑛_(𝑖, 𝑗)

𝑖=1 𝑗=1

### 108. Distance-zone entropy (DZE)

𝑁𝑔 𝑁𝑑

𝐷𝑍𝐸 = ∑ ∑ 𝑝_𝑛_(𝑖, 𝑗)log_2_[ 𝑝_𝑛_(𝑖, 𝑗)]

𝑖=1 𝑗=1

## Neighborhood Grey tone difference matrix based features

Neighborhood Grey tone difference matrix (NGTDM) based features, as described by Amadasun and

King [13]. The 𝑖th entry of the NGTDM 𝑠(𝑖|𝑑) is the sum of Grey level differences of voxels with intensity 𝑖 and the average intensity 𝐴_𝑖_ of their neighboring voxels within a distance 𝑑. In contrast to the original paper, a complete neighborhood is not required and 𝐴_𝑖_ is determined over the valid voxels. Let:

𝑛_𝑖_ be the number of voxels with Grey level 𝑖,

𝑁_𝑣_ = ∑ 𝑛_𝑖_, the total number of voxels (defined as 𝑛^2^ by Amadasun and King [13]),

𝑠(𝑖) = {∑^𝑛𝑖|𝑖 − 𝐴𝑖| for⁡𝑛𝑖 > 0^, generalized for any distance 𝑑,

0 otherwise⁡

𝑁_𝑔_ be the maximum discrete intensity level in the image,

𝑛^𝑖^

𝑝(𝑖) = , the probability of Grey level 𝑖,

𝑁_𝑣_

𝑁_𝑝_, the total number of Grey levels present in the image.

### 109. Coarseness

1

𝑐𝑜𝑎𝑟𝑠𝑒𝑛𝑒𝑠𝑠 =⁡


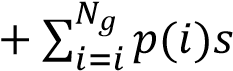
(𝑖)

Where is a small number to prevent coarseness becoming infinite.

### 110. Contrast

𝑁𝑔 𝑁𝑔 𝑁𝑔

𝑐𝑜𝑛𝑡𝑟𝑎𝑠𝑡𝑠(𝑖))


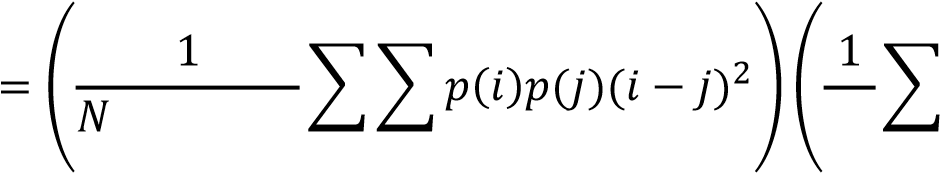
 𝑝(1 − 𝑁_𝑝_) 𝑁_𝑣_

𝑖=1 𝑗=1 𝑖=𝑖

### 111. Busyness

𝑁^𝑔^

∑ 𝑝(𝑖)𝑠(𝑖)

𝑏𝑢𝑠𝑦𝑛𝑒𝑠𝑠 = _𝑁 𝑁_^𝑖=𝑖^ , 𝑝(𝑖) ≠ 0,⁡⁡⁡𝑝(𝑗) ≠ 0


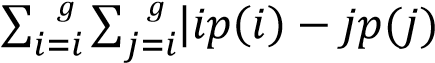
|

### 112. Complexity

𝑁𝑔 𝑁𝑔


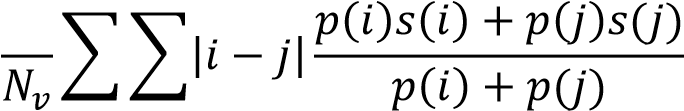
1

𝑐𝑜𝑚𝑝𝑙𝑒𝑥𝑖𝑡𝑦 =, 𝑝(𝑖) ≠ 0,⁡⁡⁡𝑝(𝑗) ≠ 0

𝑖=𝑖 𝑗=𝑖

### 113. Strength

∑𝑖𝑁=𝑔𝑖 ∑𝑁𝑗=𝑔𝑖[𝑝(𝑖) + 𝑝(𝑗)](𝑖 − 𝑗)2


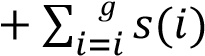
 𝑠𝑡𝑟𝑒𝑛𝑔𝑡ℎ = _𝑁_ , 𝑝(𝑖) ≠ 0,⁡⁡⁡𝑝(𝑗) ≠ 0

## Neighboring Grey level dependence matrix based features

Neighboring Grey level dependence matrix (NGLDM) based features, as described by Sun and Wee [14]. NGLDM features are invariant under spatial rotation. The (𝑖, 𝑗)th entry of the NGLDM 𝑝(𝑖, 𝑗|𝑑, 𝑎) describes the number of neighborhoods with center voxel Grey-level (i.e. intensity value) 𝑖 and dependence (i.e. number of dependent voxels) 𝑘 = 𝑗 − 1. A neighborhood are all voxels within a distance 𝑑 from the center voxel. The center voxel and a neighboring voxel are dependent if their absolute Grey value difference ≤ 𝑎, the dependency coarseness parameter. The features originally specified by Sun and Wee are analogous to the GLRLM and GLSZM features, and the feature set is extended accordingly. Let:

𝑝(𝑖, 𝑗) be the (𝑖, 𝑗)th entry in the given NGLDM 𝑝, generalized for any 𝑑 and 𝑎,

𝑁_𝑔_ the number of discrete intensity values in the image,

𝑁_𝑑_ the maximum dependence value,

𝑁_𝑠_ the total number of neighborhoods, where 𝑁_𝑠_
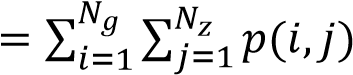
,

𝑝_𝑑_ the sum distribution of the number of neighborhoods with dependence 𝑗 = 𝑘 + 1, where 𝑝_𝑑_(𝑗) =


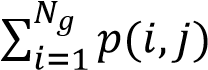
,

𝑝_𝑔_ the sum distribution of the number of neighborhoods with center voxel Grey level 𝑖, where 𝑝_𝑔_(𝑖) =


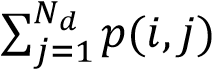
,

𝑝(𝑖,𝑗)

𝑝_𝑛_(𝑖, 𝑗) the normalized NGLDM, where 𝑝_𝑛_(𝑖, 𝑗)⁡= _𝑁_𝑠 ,

𝜇_𝑑_ the mean dependence, where 𝜇_𝑟_
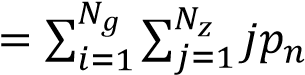
(𝑖, 𝑗|𝜃), 𝜇_𝑔_ the mean Grey level, where 𝜇_𝑔_
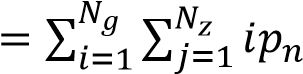
(𝑖, 𝑗|𝜃).

Note: By definition, the number of voxels in the image (𝑁_𝑝_) equals the total number of neighborhoods

(𝑁_𝑠_), since in our implementation every voxel is considered to have a neighborhood. Feature “dependence

𝑁^𝑠^ percentage” ( ), which is the equivalent to run-length feature “run percentage” (RP; 67), is therefore

𝑁𝑝

omitted, because it will always evaluate to 1.

#### 114. Small Dependence Emphasis (SDE)

𝑁𝑑

1 𝑝_𝑑_

𝑆𝐷𝐸 = 𝑁_𝑠_ ∑ 𝑗_2_

𝑗=1

This feature is also called Small Number Emphasis [14].

#### 115. Large Dependence Emphasis (LDE)

𝑁𝑑


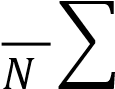
 𝐿𝐷𝐸 = 1 𝑗2𝑝𝑑

𝑠

𝑗=1

This feature is also called Large Number Emphasis [14].

#### 116. Grey-level Non-Uniformity (GLN)

𝑁𝑔

𝐺𝐿𝑁
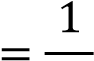
 ∑ 𝑝_𝑔_^2^

𝑁_𝑠_ 𝑖=1

#### 117. Grey-level Non-Uniformity Normalized (GLNN)

𝑁𝑔

𝐺𝐿𝑁𝑁


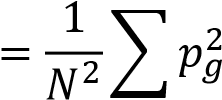
𝑠

𝑖=1

#### 118. Dependence Non-Uniformity (DN)

𝑁𝑑

𝐷𝑁𝑝_𝑧_^2^


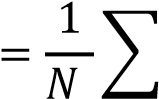
𝑠

𝑗=1

This feature is also called Number Nonuniformity [14].

#### 119. Dependence Non-Uniformity Normalized (DNN)

𝑁_𝑧_

𝐷𝑁𝑁
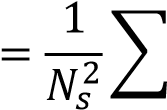
𝑝_𝑧_^2^

𝑗=1

#### 120. Low Grey-level Emphasis (LGE)

𝑁𝑔

1 𝑝_𝑔_

𝐿𝐺𝐸 = 𝑁_𝑠_ ∑ 𝑖_2_

𝑖=1

#### 121. High Grey-level Emphasis (HGE)

𝑁𝑔

𝐻𝐺𝐸
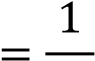
 ∑ 𝑖^2^𝑝_𝑔_

𝑁_𝑠_ 𝑖=1

#### 122. Low Grey-level small Dependence Emphasis (LGSDE)

𝑁𝑔 𝑁𝑑

1 𝑝(𝑖, 𝑗) 𝐿𝐺𝑆𝐷𝐸
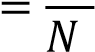
𝑠 ∑ ∑ 𝑖_2𝑗_2

𝑖=1 𝑗=1

#### 123. High Grey-level small Dependence Emphasis (HGSDE)

𝑁𝑔 𝑁

𝑑 2 𝐻𝐺𝑆𝐷𝐸
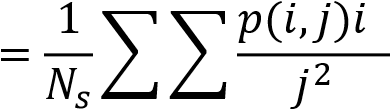


𝑖=1 𝑗=1

#### 124. Low Grey-level large Dependence Emphasis (LGLDE)

𝑁𝑔 𝑁


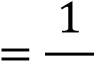

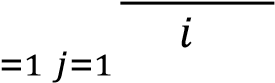
𝑑 𝑝(𝑖, 𝑗)𝑗^2^

𝐿𝐺𝐿𝐷𝐸 𝑁_𝑠_ ∑ ∑ _2_

𝑖

#### 125. High Grey-level large Dependence Emphasis (HGLDE)

𝑁𝑔 𝑁𝑑

𝐻𝐺𝐿𝐷𝐸
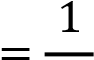
 ∑ ∑ 𝑝(𝑖, 𝑗)𝑖^2^𝑗^2^

𝑁_𝑠_

𝑖=1 𝑗=1

#### 126. Grey-level variance (GLV)

𝑁𝑔 𝑁𝑑

𝐺𝐿𝑉 = ∑ ∑(𝑖 − 𝜇_𝑔_)^2^𝑝_𝑛_(𝑖, 𝑗)

𝑖=1 𝑗=1

#### 127. Dependence variance (DV)

𝑁𝑔 𝑁𝑑

𝐷𝑉 = ∑ ∑(𝑗 − 𝜇_𝑑_)^2^𝑝_𝑛_(𝑖, 𝑗)

𝑖=1 𝑗=1

#### 128. Dependence entropy (DE), also called Entropy [14]

𝑁𝑔 𝑁𝑑

𝐷𝐸 = ∑ ∑ 𝑝_𝑛_(𝑖, 𝑗)log_2_[ 𝑝_𝑛_(𝑖, 𝑗)]

𝑖=1 𝑗=1

Note: the definition of entropy by Sun and Wee [14] uses the dependence counts (𝑝) instead of the dependence propabilities (𝑝_𝑛_).

#### 129. Second moment (SM) [14]

𝑁𝑔 𝑁𝑑 𝑝(𝑖, 𝑗)2

∑𝑖=1 ∑𝑗=1

𝑆𝑀 =

𝑁_𝑠_

Note: for this feature, defined by Sun and Wee [14], there is no Grey-level run-length equivalent

## Wavelet features

Wavelet transform effectively decouples textural information by decomposing the original image, in a similar manner as Fourier analysis, in low– and high-frequencies. In this study a discrete, one-level and undecimated three dimensional wavelet transform was applied to each CT and CBCT image, which decomposes the original image 𝑋 into 8 decompositions. Consider 𝐿 and 𝐻 to be a low-pass (i.e. a scaling) and, respectively, a high-pass (i.e. a wavelet) function, and the wavelet decompositions of 𝑋 to be labeled

as 𝑋_𝐿𝐿𝐿_, 𝑋_𝐿𝐿𝐻_,⁡𝑋_𝐿𝐻𝐿_,⁡𝑋_𝐿𝐻𝐻_,⁡𝑋_𝐻𝐿𝐿_,⁡𝑋_𝐻𝐿𝐻_, 𝑋_𝐻𝐻𝐿_ and⁡𝑋_𝐻𝐻𝐻_. For example, 𝑋_𝐿𝐿𝐻_ is then interpreted as the high-pass sub band, resulting from directional filtering of 𝑋 with a low-pass filter along x-direction, a low pass filter along y-direction and a high-pass filter along z-direction and is constructed as:

𝑁𝐿 𝑁𝐿 𝑁𝐻

𝑋_𝐿𝐿𝐻_(𝑖, 𝑗, 𝑘) = ∑ ∑ ∑ 𝐿(𝑝)𝐿(𝑞)𝐻(𝑟)𝑋(𝑖 + 𝑝, 𝑗 + 𝑞, 𝑘 + 𝑟)

𝑝=1 𝑞=1 𝑟=1

Where 𝑁_𝐿_ is the length of filter 𝐿 and 𝑁_𝐻_ is the length of filter 𝐻. The other decompositions are constructed in a similar manner, applying their respective ordering of low or high-pass filtering in x, y and z-direction. Wavelet decomposition of the image 𝑋 is schematically depicted in Figure 7. Since the applied wavelet decomposition is undecimated, the size of each decomposition is equal to the original image and each decomposition is shift invariant. Because of these properties, the original tumor delineation of the gross tumor volume (GTV) can be applied directly to the decompositions after wavelet transform. In this study “Coiflet 1” wavelet was applied. For each decomposition we computed the first order gray level statistics and the textural features (GLCM, GLRLM, GLSZM).


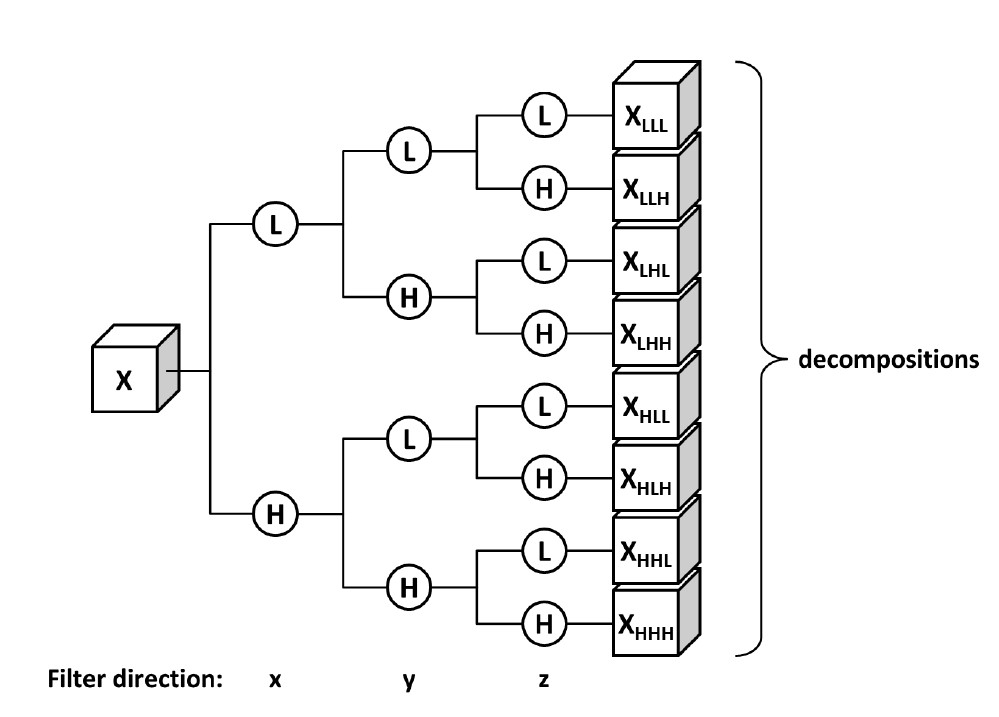


Figure 8: Schematic of the undecimated three dimensional wavelet transform applied to each CT/CBCT image. The original image 𝑋 is decomposed into 8 decompositions, by directional low-pass (i.e. a scaling) and high-pass (i.e. a wavelet) filtering:

𝑋𝐿𝐿𝐿, 𝑋𝐿𝐿𝐻,⁡𝑋𝐿𝐻𝐿,⁡𝑋𝐿𝐻𝐻,⁡𝑋𝐻𝐿𝐿,⁡𝑋𝐻𝐿𝐻, 𝑋𝐻𝐻𝐿 and⁡𝑋𝐻𝐻𝐻.

## Laplacian of Gaussian features

The Laplacian of an image brings out areas of rapid intensity change and is usually used for edge detection. A Gaussian filter is applied prior to the Laplacian to smooth the image and reduce noise. Textural properties representing features of different degrees of coarseness can then be calculated. The equation of a LoG with a 2D kernel:

LoG(x, y) = − 1 [1 − x22+σ_2_y2] e−x22+σy22


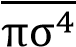


Texture size (fine to coarse) is highlighted by modifying the Gaussian radius parameter 𝜎 (e.g., from 0.5 mm to 5mm, with 0.5 mm increments). Each value of 𝜎 provides a filtered image. First-order gray-level statistics (described earlier) are determined for each filtered image, as well as for only the positive part of each filtered image.

# References

1. Feldkamp LA, Davis LC, Kress JW. Practical cone-beam algorithm. Journal of the Optical Society of America A 1984;1(6):612-619.
2. Aerts HJ, Velazquez ER, Leijenaar RT*, et al.* Decoding tumour phenotype by noninvasive imaging using a quantitative radiomics approach. Nat Commun 2014;5:4006.
3. Sousa JR, Silva AC, de Paiva AC*, et al.* Methodology for automatic detection of lung nodules in computerized tomography images. Computer methods and programs in biomedicine 2010;98(1):1-14.
4. Haralick RM, Shanmugam K, Dinstein I. Textural Features of Image Classification. IEEE Transactions on Systems, Man and Cybernetics 1973;SMC-3(6):610-621.
5. Clausi DA. An analysis of co-occurrence texture statistics as a function of grey level quantization. Canadian Journal of remote sensing 2002;28(1):45-62.
6. Tsatsoulis LSaC. Texture Analysis of SAR Sea Ice Imagery Using Gray Level Co-Occurrence Matrices. IEEE Transactions on Geoscience and Remote Sensing 1999;37(2).
7. Galloway M. Texture analysis using gray level run lengths. Computer Graphics and Image Processing 1975;4:172-179.
8. Albregtsen F, Nielsen B, Danielsen HE. Adaptive gray level run length features from class distance matrices. In: *Pattern Recognition, 2000. Proceedings. 15th International Conference on*, *2000*: Abstract 3, p. 738-741 vol.3.
9. Thibault GF, B; Navarro, C; Pereira, S. Texture indexes and gray level size zone matrix: application to cell nuclei classification. Pattern Recognition Inf Process. 2009:140-145.
10. Tixier F, Hatt M, Le Rest CC*, et al.* Reproducibility of tumor uptake heterogeneity characterization through textural feature analysis in 18F-FDG PET. Journal of nuclear medicine : official publication, Society of Nuclear Medicine 2012;53(5):693-700.
11. Tixier F, Le Rest CC, Hatt M*, et al.* Intratumor heterogeneity characterized by textural features on baseline 18F-FDG PET images predicts response to concomitant radiochemotherapy in esophageal cancer. Journal of nuclear medicine : official publication, Society of Nuclear Medicine 2011;52(3):369-378.
12. Thibault G, Angulo J, Meyer F. Advanced Statistical Matrices for Texture Characterization: Application to Cell Classification. IEEE Transactions on Biomedical Engineering 2014;61(3):630-637.
13. Amadasun M, King R. Textural features corresponding to textural properties. Systems, Man and Cybernetics, IEEE Transactions on 1989;19(5):1264-1274.
14. Sun C, Wee WG. Neighboring gray level dependence matrix for texture classification. Computer Vision, Graphics, and Image Processing 1983;23(3):341-352.
